# Cytokine‐Mediated Responses Across Endplate Subregions in Intervertebral Disc Multi‐Tissue Explants

**DOI:** 10.1002/jsp2.70225

**Published:** 2026-11-11

**Authors:** Ahmad Alminnawi, Pablo Tejero, Carla Geeroms, Liesbet Geris, Christine L. Le Maitre

**Affiliations:** ^1^ Prometheus, Division of Skeletal Tissue Engineering KU Leuven Leuven Belgium; ^2^ GIGA In Silico Medicine University of Liège Liège Belgium; ^3^ Biomechanics Section KU Leuven Leuven Belgium; ^4^ School of Medicine and Population Health University of Sheffield Sheffield UK

**Keywords:** bony endplate, cartilaginous endplate, endplate, extracellular matrix (ECM), glycosaminoglycan (GAG), interleukin‐1 beta (IL‐1β), intervertebral disc, tumor necrosis factor (TNF)

## Abstract

**Background:**

The endplate (EP) is essential for maintaining intervertebral disc (IVD) homeostasis by regulating nutrient transport to the nucleus pulposus (NP). Structural or biochemical alterations in the EP can impair this function and contribute to disc degeneration and low back pain (LBP). While most studies focus on the NP and annulus fibrosus (AF), increasing evidence suggests that early EP degeneration is a key initiator of disc pathology.

**Aims:**

This study combined *in silico* modeling with a novel ex vivo multi‐tissue culture system to investigate cytokine‐driven degeneration across the intact IVD‐vertebral interface. A previously validated signal‐transduction network model predicted that degenerative environmental conditions alone, without cytokine stimulation, are limited in inducing chondrocyte degeneration.

**Materials & Methods:**

To experimentally corroborate this, intact bovine explants comprising NP, AF, cartilaginous EP, and bony EP were maintained in a composite culture system, preserving tissue interfaces, an approach not previously reported. Explants were cultured for 14 days under standard, degenerative, and cytokine‐stimulated conditions using tumor necrosis factor (TNF) or interleukin‐1‐beta (IL‐1β). Tissue responses were assessed using computed tomography, glycosaminoglycan‐to‐DNA (GAG/DNA) ratios, GAG release, histological grading, cell density, and immunohistochemistry.

**Results:**

*In silico* predictions were supported experimentally, whereby degenerative conditions alone induced limited changes associated with degeneration, whereas cytokine exposure induced broader and more pronounced degenerative changes. Reduced proteoglycan staining in the CEP and NP/AF tissues was seen under cytokine conditions without complete structural disruption. Furthermore, staining for anabolic and catabolic markers in the CEP was altered within 14 days.

**Discussion & Conclusion:**

These findings suggest that cytokine signaling is an important driver and amplifier of EP‐associated degeneration with distinct, tissue‐specific responses. The development of a multi‐tissue ex vivo culture system provides a novel platform to study degenerative mechanisms under physiologically relevant investigation of tissue interactions.

## 1 Introduction

Low back pain (LBP) affects approximately 80% of individuals in Western countries during their lifetime, accounting for nearly 5 million disability‐adjusted life years (DALYs) in young adults, making it the leading cause of morbidity worldwide [1, 2, 3]. Among the various components of the spine, the endplate (EP) has received comparatively little attention in the scientific literature [4], despite its critical role in maintaining the health of the intervertebral disc (IVD). Whilst calcification‐induced reduction in EP permeability leads to decreased nutrient supply to the disc [5, 6], matrix degradation and microfissures within the EPs facilitate infiltration of blood vessels and nerve fibers from the subchondral bone into the otherwise immune‐privileged IVD^1^. This makes EP changes a principal contributing factor in LBP [7, 8].

The EP is a layered composite structure consisting of the bony endplate (BEP), a semiporous region of thickened cancellous bone that is part of the vertebral body (VB), and the cartilaginous endplate (CEP). The CEP is subdivided into the non‐calcified (ncCEP) region, associated with the IVD, and the calcified (cCEP) region, which serves as a transition between the VB and IVD. As a thin layer of hyaline‐like cartilage, the CEP separates the IVD from the VB [4, 9, 10], acting both as a mechanical barrier that prevents the nucleus pulposus (NP) from bulging into the VB and proteoglycans from leaching out [11], and is the principal conduit for nutrient and waste transport to sustain the avascular NP [4, 11].

Cytokines, particularly tumor necrosis factor (TNF) and interleukin‐1 beta (IL‐1β) [12, 13, 14], are key mediators of catabolic processes in cartilage and IVD and are upregulated in degenerating tissue. These cytokines are produced by resident chondrocytes within the CEP, and the NP and annulus fibrosus (AF) cells throughout the IVD [15, 16], and drive matrix degradation via stimulation of matrix‐degrading enzymes. In the IVD, elevated TNF and IL‐1β correlate with increased degeneration, whereas healthy tissues show low levels of IL‐1β, which is balanced by expression of its natural antagonist IL‐1Ra, whilst TNF shows minimal expression. IL‐1β and TNF have been implicated in disc degeneration and herniation, promoting catabolic activity, neurotrophic factor expression, and altered matrix synthesis [13, 16, 17, 18, 19]. However, within the native human IVD, IL‐1β appears to play a greater role due to limited TNF receptor expression in vivo [12, 20]. Yet, the majority of studies to date have focused on the response of NP cells and tissues to these cytokines [21, 22, 23, 24, 25, 26], with some studies on AF cells and tissues [24, 26, 27, 28], but limited work to date has investigated the response and roles of these cytokines within the EPs [4, 29, 30]. In particular, the specific regulatory roles of TNF and IL‐1β within the EP subregions remain poorly defined, with no studies to date investigating intact multi‐tissue explants. Notably, most EP experimental models rely on isolated cell cultures or single‐tissue explants, which fail to capture the structural and functional coupling between the IVD, CEP, and BEP. This isolation does not adequately represent its in vivo function. Interactions across this multi‐tissue interface are likely to play a central role in the initiation and progression of degeneration, particularly in response to cytokine stimuli. However, to date, no studies have investigated cytokine‐driven EP degeneration in an intact multi‐tissue IVD‐VB system that preserves these native interfaces.

Human tissue studies have provided important evidence that the EP is an active component of IVD degeneration and that the CEP interacts with adjacent disc tissues. The work of Dudli et al. provided evidence of biological cross‐talk between the IVD, EP, and adjacent vertebral bone marrow in the context of Modic changes [31]. This cross‐talk was hypothesized as early as 1976 when Neidlinger‐Wilke et al. demonstrated that conditioned media from human CEP tissue could induce inflammatory and catabolic responses in NP cells [32], providing direct evidence that the CEP can modulate disc cell behavior. However, human tissues are inherently heterogeneous, with variation in donor age, degeneration severity, tissue composition, and comorbidities, while intact matched multi‐tissue specimens are difficult to obtain. Experimental animal studies have further demonstrated that EP injuries or pathological stimuli can promote degenerative and inflammatory responses across the IVD‐EP‐VB interface [33, 34, 35]. Together, these findings highlight the importance of studying the IVD and EP as an integrated system while supporting the need for a controlled, reproducible multi‐tissue model that preserves these native interfaces.

Therefore, this study developed a novel ex vivo explant culture model that maintains the IVD‐VB architecture, including NP/AF, CEP, and BEP. This platform enables, for the first time, the investigation of spatially resolved, cytokine‐mediated degenerative responses across the EP subregions within an integrated multi‐tissue environment.

## 2 Methods

### 2.1 Predictive Computational Model of Chondrocyte Degeneration With and Without Cytokines

Chondrocyte degeneration was assessed *in silico* in degenerative media with and without cytokines using a previously validated *in silico* signal‐transduction network model developed for cartilage degeneration in osteoarthritis [36, 37]. The resulting chondrocyte states were compared against reference profiles representing healthy and degenerative phenotypes. The model integrated coupled gene regulatory and protein signaling subnetworks, comprising 74 normalized parameters (0–1 scale) that collectively represent approximately 300 direct and indirect biochemical interactions governing cellular behavior. Model outputs included gene‐level (slow) and protein‐level (fast) activities, with global activity defined as their product [36, 37, 38].

The analysis was performed using a Monte Carlo approach with 10 000 random initializations, each of which was ran until a stable steady state was reached. Of these steady states, 11 were classified as healthy and subsequently used as the initial conditions for the simulations. These 11 healthy initial states were perturbed by clamping selected nodes to values reflecting in vitro conditions for 1000‐time steps. After the clamp was released, the model was simulated until a new steady state was reached. To account for the stochasticity of the model, each perturbation was performed in 100 independent runs for each of the 11 initial states, resulting in 1100 runs for each environment. Although degenerative conditions differ from healthy conditions in both osmolarity and pH [39], the latter was not incorporated as a variable in the *in silico* model because there was insufficient quantitative experimental data to reliably parameterize and validate its effects on the signaling network. Nevertheless, pH was included in the subsequent in vitro experiments to address this knowledge gap and provide evidence that may support the incorporation of pH into future in silico models. Therefore, degenerative conditions without cytokines were simulated as low osmolarity conditions, captured in the model through overexpression of TRPV4 (Transient Receptor Potential cation channel subfamily V member 4), based on in‐house experiments and literature reports [40, 41, 42]. Degenerative conditions with cytokines additionally included the overexpression of the generic cytokine variable in the model.

Following clamp release, the system evolved to a new steady state and was classified by comparison against predefined healthy and degenerate (hypertrophic‐like disease) reference attractor states. The degenerate states are defined as having high global activity of RUNX2, COL‐X, MMP13, and IHH, along with low global activity of SOX9, NKX3.2, COL‐II, ACAN, and proteoglycans, whereas healthy states exhibited the opposite activity profile as previously reported by our group [36, 37]. Simulation code is available at http://www.biomech.ulg.ac.be/software/.

### 2.2 Bovine Disc Model

The bovine IVD is increasingly recognized as a valuable model for studying the mechanobiology, cell biology, and pathogenesis of human IVD degeneration, owing to anatomical and biochemical similarities in cell phenotype, composition, and mechanical properties [43]. Research indicates that bovine CEP closely resembles that of humans in composition and organization, including proteoglycan distribution and gene expression profiles [44], in addition to responses to cytokines, particularly TNF and IL‐1β [45, 46, 47]. In view of these similarities and the ethical and practical limitations of obtaining healthy, intact human VB‐IVD samples, bovine tails were used to study the cytokine effects on the CEP over a 14‐day experiment in either standard disc culture media or media modulated to mimic the degenerate environment [39] (Figure 1).

**FIGURE 1:**
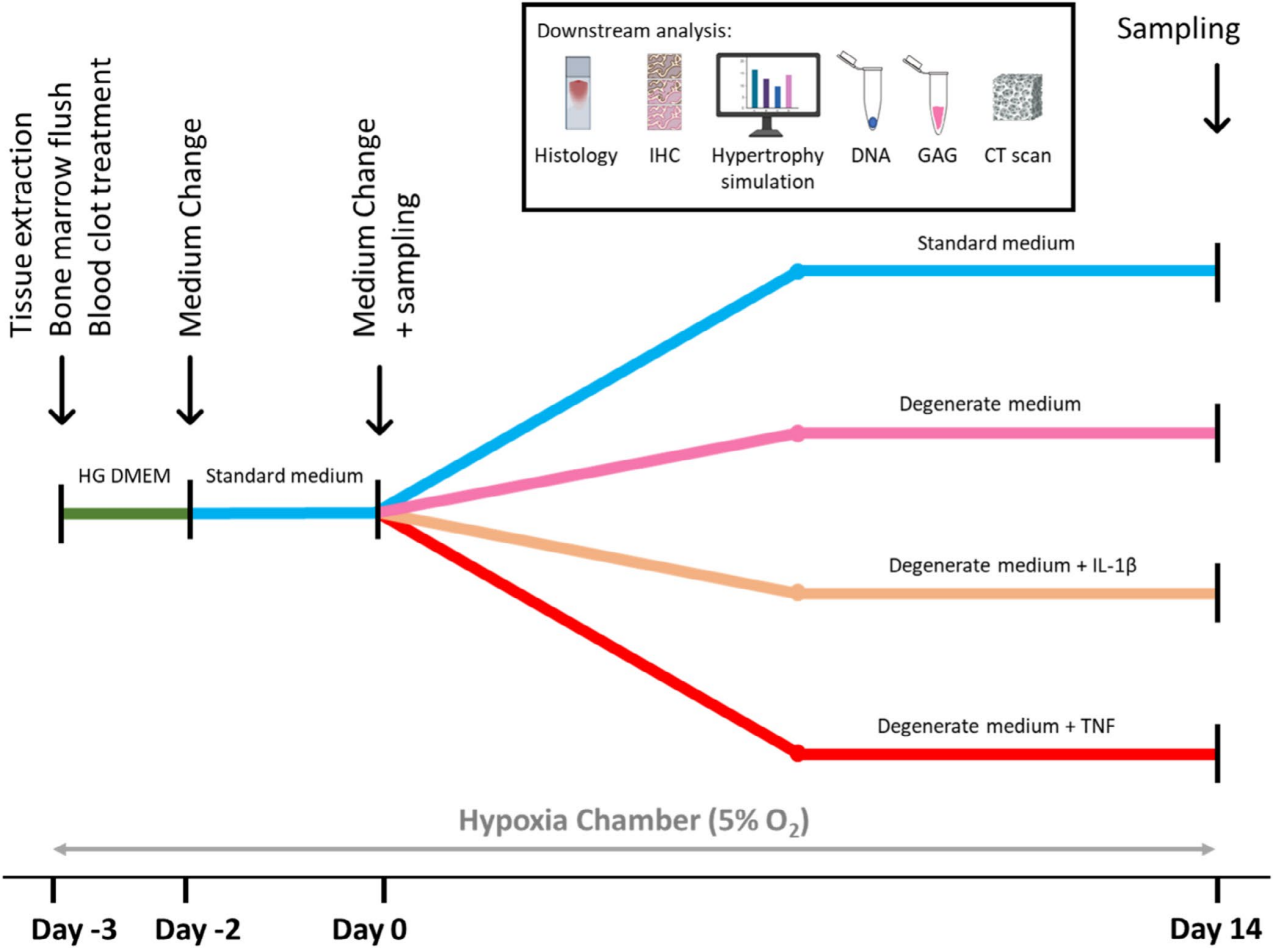
Chronological overview of the experimental procedures from the tissue extractions at day −3 to the sampling and storing at days 0 and 14, and the downstream analysis performed subsequently. Standard and degenerate media are made of LG‐DMEM supplemented with FCS, with cytokine‐treated groups denoted by “+ TNF” or “+ IL‐1β.” More details for the media composition can be found in Table 2.

### 2.3 Tissues Extraction and Isolation

The tissue harvesting protocol was adapted from published methodology [48, 49] where three fresh whole‐length bovine tails were acquired from the local abattoir (Bramall & Son, Sheffield). The tails were acquired without skin to reduce the chance of contamination and immediately sterilized with betadine solution and placed under the laminar flow to be processed. Muscles, ligaments, and outer connective tissues were dissected to expose the vertebrae and IVDs, and around 10 intact IVDs with 8.4 ± 2.6 mm of adjacent vertebrae on each side were excised from the middle section of each tail. These IVDs were then washed 3–6 times with PBS (Phosphate Buffer Saline) to remove the bone marrow and any blood clots. The vertebral‐IVD explants were then cut using a veterinarian oscillating bone saw (NS‐1011 Oscillating Bone Saw Medical from Wuhu Ruijin Medical Instrument and Device Co. Ltd.). A total of 91 samples were harvested across three bovine tails (Table 1). Samples were randomly assigned to the different experimental groups (Supplementary Table S.1). The IVD tissue side of each of the samples was then dipped in sterile 3% w/v agarose prepared in PBS to reduce glycosaminoglycans (GAG) depletion and tissue swelling [50].

**TABLE 1:** Number of explants obtained per bovine stratified per environment.

Day	Treatment	Test	Bovine 1	Bovine 2	Bovine 3
0	Day 0	GAG/DNA	3	3	—
		CT—histology	3	3	—
14	Standard	GA G/DNA	4	4	3
		CT—histology	3	3	3
	Degenerate	GAG/DNA	3	3	3
		CT—histology	3	3	3
	Degenerate + IL‐1β	GAG/DNA	4	4	3
		CT—histology	3	4	3
	Degenerate + TNF	GAG/DNA	3	4	3
		CT—histology	3	4	3
Total: 91			32	35	24

*Note:* At day 0 (3 days post‐harvesting) and standard, degenerate, degenerate + IL‐1β, or degenerate + TNF at day 14.

### 2.4 Culture Conditions

Whereas the in silico model compared healthy and degenerative environments by altering osmolarity, the experimental study compared standard and degenerative culture conditions. This approach was chosen because tissue culture studies commonly use standard culture conditions as the reference group [41, 42] rather than attempting to recreate a fully healthy intervertebral disc environment, facilitating comparison with existing literature. Furthermore, the objective of the experimental work was to evaluate tissue‐level responses rather than isolated cell behavior. Sample availability was limited, which precluded the inclusion of an additional healthy experimental group.

The basal media for all cultures was DMEM (Dulbecco's Modified Eagle Medium, Gibco), supplemented with 10% v/v FCS (Fetal Bovine Serum, Gibco: A5256701). During the initial 24 h, all samples were placed in a 5% oxygen hypoxia glove box (Don Whitley Hypoxia station) with standard high‐glucose medium (4.5 g/L). This step allowed the cells and tissues to recover from mechanical disruption during harvesting and sudden loss of native nutrient supply. Subsequently, the medium was switched to standard low‐glucose (1 g/L) DMEM to prevent high‐glucose‐induced damage to CEP cells [51] and maintain a more physiological glucose level. Culture under low glucose and 5% O_2_ was maintained for 48 h before the samples were allocated randomly to experimental conditions. Following the initial 3 day pre‐culture to enable tissues to recover from extraction, this was defined as ‘day 0’ of the experiment and *n* = 6 samples from bovine 1 and bovine 2 were taken for downstream analysis as “day 0 controls” as described later, while all remaining samples were split into 4 groups: standard medium, degenerate medium, degenerate + IL‐1β medium, and degenerate + TNF medium (Table 2). The key difference between the standard and the degenerate media is the pH levels that were imparted with the differences in the Sodium Bicarbonate concentrations [39]. Both standard and degenerate media had an OSM of 325 mOsm/Kg, whilst the pH of the standard medium was 7.2 and of the degenerate media was 6.8, as reported recently for NP media [39]. All cultures were performed in 6‐well plates filled with 10 mL of the designated medium, and swelling was minimized via the application of the agarose “dip.” All media were stored and refreshed three times per week until day 14, after which time all remaining samples were collected for downstream analysis.

**TABLE 2:** Tissue culture media used throughout the experiment, where high‐glucose was used for freshly harvested samples from day −3 till day −2, and standard media applied from day −2 to day 0, and standard, degenerate, and degenerate with cytokines were used from day 0 till day 14. DMEM.

Component	Amount (Final concentration)	Supplier and product code
High‐glucose medium		
HG‐DMEM	(500 mL) As supplied	Gibco: 10569010
Pen/Strep	(50 U/mL)	Gibco: 15070‐063
Amphotericin B	(2.5 μg/mL)	Sigma: A2942
L‐Ascorbic acid 2‐phosphate sesquimagnesium salt hydrate	(50 μg/mL)	Sigma: A5960
FCS	10% v/v	Gibco: A5256701
Standard medium		
LG‐DMEM	(500 ml) as supplied	Gibco: 11885084
Pen/Strep	(50 U/mL)	Gibco: 15070–063
Amphotericin B	(2.5 μg/mL)	Sigma: A2942
L‐Ascorbic acid 2‐phosphate sesquimagnesium salt hydrate	(50 μg/mL)	Sigma: A5960
FCS	10% v/v	Gibco: A5256701
Degenerate media		
LG‐DMEM powder	4.99 g per 500 mL	Gibco: 31600–083
Sodium bicarbonate	0.213 g per 500 ml	Sigma: S‐6014
Pen/Strep	(50 U/mL)	Gibco: 15070–063
Amphotericin B	(2.5 μg/mL)	Sigma: A2942
L‐Ascorbic acid 2‐phosphate sesquimagnesium salt hydrate	(25 μg/mL)	Sigma: A5960
FCS	10% v/v	Gibco: A5256701
L‐Glutamine	1% v/v	Gibco: 25030–024
IL‐1β	1 ng/mL (for degenerate + IL‐1β medium)	Peprotech (200‐01B‐10UG)
TNF	1 ng/ml (for degenerate + TNF medium)	Peprotech (300‐01A‐50UG)

## 3 Downstream Analysis

At day 0 and at day 14, samples were split into two groups; one group was fixed using 4% w/v formaldehyde (Sigma Aldrich 1.04003) and stored at room temperature prior to imaging using a μCT (micro computed tomography) scanner. Following CT analysis, samples were EDTA decalcified in 20% w/v EDTA/TBS and embedded in paraffin wax and processed for histology and immunohistochemistry to study the microscopic anatomy of biological tissues. Samples of the second group were separated into their component tissue parts (VB, CEP, IVD) and snap frozen in liquid nitrogen, then stored at −80°C for GAG (glycosaminoglycans) and DNA (Deoxyribonucleic acid) quantification. Additionally, the spent media from the technical replicates were pooled for each bovine subject and stored at −80°C to calculate the GAGs released by the cells/tissues into the media.

### 3.1 CT Scanning

Although it is difficult to display the structure of the CEP, NP, and AF on CT scans due to its poor contrast in soft tissues [52], the high resolution provided by the Phoenix Nanotom by Baker Hughes (Waygate Technologies), in addition to short 3D scanning time, enabled a detailed examination of the bone tissue integrity and the detection of any boundary artifacts. To better visualize the non‐calcified cartilage in the explants, Hexabrix was used as a contrast agent since it interacts electrostatically with the negatively charged GAGs present in cartilage and IVD [53, 54]. The formaldehyde‐fixed samples were submerged and incubated in 40% Hexabrix for 48 h at room temperature, enabling imaging of the IVD [55]. The samples were then wrapped with parafilm and fitted into holders to ensure they were fixed in place while being imaged in the CT scanner using in‐house optimized settings (Table 3), for osteochondral tissues with a resolution of 9 μm. The exposure time was 500 ms, and 1800 images were acquired over 360°. The free and open source software, 3D Slicer 5.0.3 [56, 57], was used to visualize the scans and analyze them. Defects in the CEP‐BEP interface were noted, and the maximum thicknesses of the BEP, CEP, and soft tissues (comprising both NP and AF) were calculated in each sample.

**TABLE 3:** In‐house optimized settings of μCT scanner for osteochondral tissues standardized and used for all the explant samples.

X‐ray source parameters		Acquisition parameters		Reconstruction parameters	
Voltage	60 KV	Exposure time	0.5 s	bhc^a^	8
Current	170 μA	Voxel size	9 μm	Filter	Inline median
Filter	0.2 mm Aluminum	Fast scan	Yes		ROI CT filter
Target	Diamond‐coated Tungsten				Filter volume

^a^ bhc: beam hardening correction.

Although the endplates, especially the bony endplate regions, have been defined in different ways in the literature [58], standardized definitions were adopted for CT‐based segmentation. The soft tissues, NP/AF, were identified as the medium CT intensity regions with smooth texture at one extremity of the explant. The CEP was divided into ncCEP, characterized by very low CT intensity between bone and soft tissue, and cCEP, the highest intensity layer separating the IVD from the VB; these two regions were separated by the tidemark, and the cCEP was separated from the BEP by the cement line. The BEP and subchondral trabecular bone (STB) showed similar intensities, but STB exhibited larger, marrow‐filled pores. The epiphyseal growth plate, present only in younger bovines and humans, appeared as an uncalcified layer separating STB from metaphyseal trabecular bone (MTB). This clear definition allowed consistent measurement of maximal thickness for each compartment (Figure 2). For subsequent analyses, “soft tissues” (Figure 2 tissue “a”) included NP and AF, “CEP” included cCEP and ncCEP (Figure 2 tissues “b” and “c” respectively), whereas the VB region comprised BEP, STB, growth plate, and MTB (Figure 2 tissues “d,” “e,” “f,” and “g,” respectively).

**FIGURE 2:**
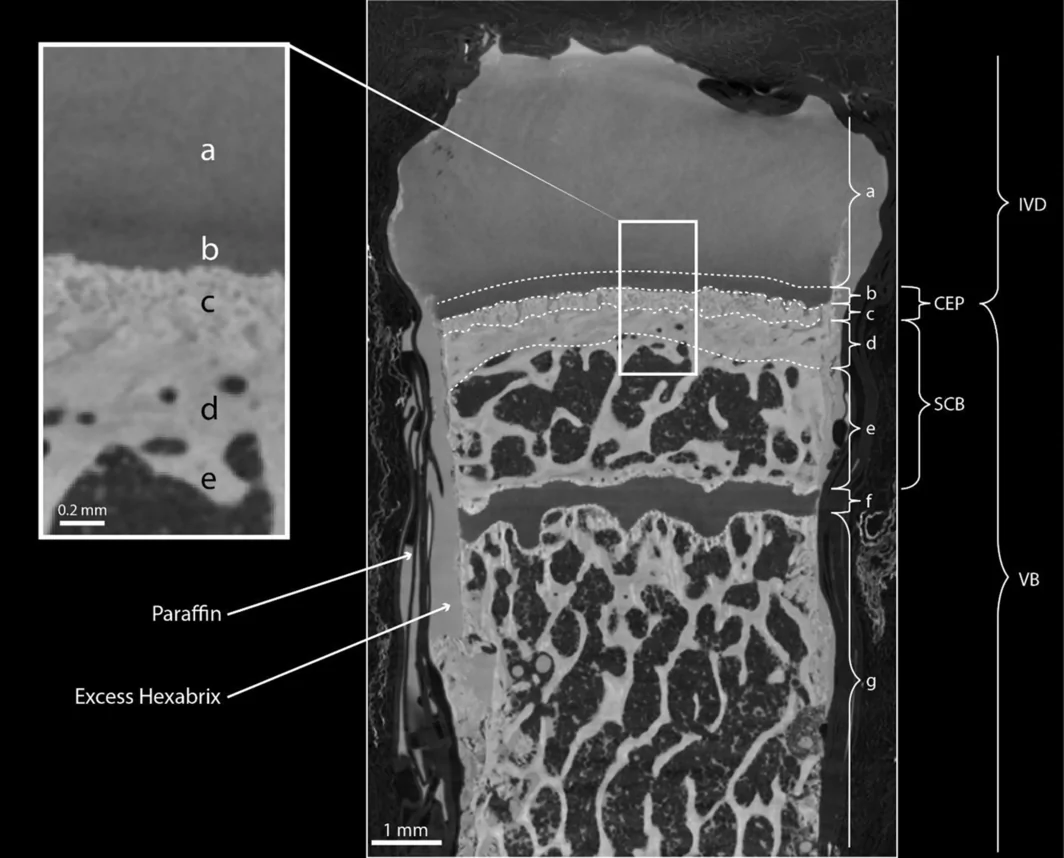
A labeled sample of the scanned explant in the μCT where lowercase letters represent the different tissues (a) nucleus pulposus and/or annulus fibrosus (NP/AF), (b) non‐calcified cartilaginous endplate (ncCEP), (c) calcified cartilaginous endplate (cCEP), (d) bony endplate (BEP), (e) subchondral trabecular bone (STB), (f) growth plate, and (g) metaphyseal trabecular bone (MTB). Scale bar = 1 mm.

### 3.2 Glycosaminoglycan (GAG) Quantification

Downstream, the frozen samples were digested in papain solution from papaya (Sigma Aldrich P3125) for 12 h at 60°C. The papain digestion solution was made using 0.31 mg/mL DTT (dithiothreitol (Thermo Scientific R0861)) and 1 unit/ml papain in buffer solution that contained 20 mM sodium phosphate monobasic (Sigma Aldrich S9638), 20 mM sodium phosphate dibasic (Sigma Aldrich S0876), and 1 mM EDTA·2H_2_O. The digested samples, in addition to the collected media, were then used separately to quantify GAG using the DMMB (Dimethylmethylene Blue (Sigma Aldrich 341 088)) assay [59] with known concentrations of standard bovine cartilage chondroitin sulphate C solution (Sigma Aldrich C6737). The absorbance of the samples and the standards was measured at 525 and 595 using an ELISA multimode plate reader (TECAN Infinite 200 PRO). The difference of absorbance was used to construct the standard curve [60] and the coefficient of determination (*R* ^2^) was calculated to assess the linear regression line, which was 0.995 for both the GAGs in the tissues and GAGs in the media. Media of samples collected from each subject were pooled by condition prior to the quantitative analysis.

### 3.3 Deoxyribonucleic Acid (DNA) Quantification

To enable normalization to DNA content, the papain‐digested samples used for GAG assessment were used to quantify DNA using the PicoGreen assay (Quant‐iT PicoGreen dsDNA Assay Kits and dsDNA Reagents, Catalog number P7589, Thermo Fisher Scientific) following the manufacturer's instructions. The fluorescence wavelengths were measured at excitation of 480 nm and emission of 520 nm on the same ELISA multimode plate reader. The standard curve was created using known concentrations of λ‐DNA stock solution, and the *R* ^2^ was calculated, which was 0.998 for DNA. The GAG/DNA ratio was then determined.

### 3.4 Histological Examination

Fixed samples were decalcified in 20% EDTA (Ethylenediaminetetraacetic Acid (Sigma Aldrich ED2SS)) for 3 months, with 2–3 solution changes per week. Samples were then embedded in paraffin (VWR International 10 048 502) and sectioned at 5 μm using a Microtome HM355S (Thermo Fisher Scientific). Sections were mounted on Superfrost Microscope Slides (AG00008032E01MNZ20, Thermo Fisher Scientific) for histological staining. Four different staining techniques were used to assess the structure and composition of the explants. The slides were dewaxed using Histo‐Clear (National Diagnostic HS‐200) and methanol before being dipped in their respective stain and counterstains: Alcian Blue/Nuclear Fast Red (AB), Safranin Orange/Fast Green (SafO), Hematoxylin/Eosin (H&E), and Masson Trichrome (MTC). For all stainings, sections were first deparaffinized in Histo‐Clear and rehydrated through graded alcohols to distilled water. For Alcian Blue staining, sections were incubated in 0.5% Alcian Blue solution (A3157 Sigma) at pH 1 for 60 min, rinsed, and counterstained with Nuclear Fast Red (642 425 Klinipath) for 5 min. For Safranin O/Fast Green staining, sections were stained with hematoxylin for 1 min, then acid alcohol for 5 s. Subsequently, sections were stained with Fast Green (Klinipath; 80 051; color index 42 053) for 5 min and 1% acetic acid for 30 s, followed by Safranin O staining (Klinipath 640 780) for 10 min. For H&E staining, sections were stained with hematoxylin (Sigma Aldrich H9627) for 5 min, washed under running water, and counterstained with eosin (Clin‐Tech 640 381) for 7 min. For Masson's Trichrome staining, sections were refixed with Bouin's solution (Polysciences 16 045) at 60°C for 1 h, stained sequentially with Weigert's Iron Hematoxylin (Sigma HT1079) for 10 min, Biebrich Scarlet red (Sigma HT151) for 5 min, a 1:1 mixture of phosphotungstic (Sigma HT152) phosphomolybdic acid and (Sigma HT153) for 10 min, and Aniline Blue (Sigma, B8563) for 5 min, followed by differentiation in 1% acetic acid for 1 min. After staining, all sections were rinsed between steps in dH_2_O, and after staining was performed, then dehydrated through graded ethanol, Histo‐Clear, and mounted with Pertex (Histolab 00801‐EX).

All samples were processed and stained on the same day using the same staining solution batches to ensure consistent conditions and comparable results. All samples were imaged using a brightfield microscope (Olympus IX83, Olympus Corporation, Tokyo, Japan) equipped with an Olympus DP73 digital camera and Olympus cellSens Dimension image acquisition software (version 1.18).

Stained area and staining intensities in AB, SafO, and MTC images were quantified using Fiji ImageJ [61]. To reduce background‐related false positives, AB and MTC images were color‐shifted to red, and SafO images to blue. Color deconvolution [62] was then performed to digitally isolate the stains using built‐in ImageJ parameters specific to each stain. The CEP was traced to define the region of interest (ROI). Binary masks were generated by applying automatic thresholding to the primary stain channel, using the Huang, Rényi entropy, and Mean methods for AB, SafO, and MTC, respectively. The stained area percentage within the ROI was measured from the binary mask, while the mean color intensity was extracted directly from the primary stain channel. The full set of algorithms covering these tools is available in a public GitHub repository [63], along with a user manual.

Since no standardized degeneration scoring system exists specifically for the bovine EP, the system used (Table 4) was an adaptation based on commonly reported parameters from several human and large animal models [64, 65, 66, 67, 68], focusing only on the CEP and BEP regions. Each sample was assigned a score from 0 to 3 for each of six criteria related to EP degeneration, where 0 indicated no degeneration, 1 indicated early/mild degeneration, 2 indicated moderate degeneration, and 3 indicated severe degeneration, resulting in a total possible degeneration score of 18. CEP cellularity was assessed based on clustering, as well as features of neovascularization. Cellular degeneration was evaluated by identifying hallmarks of apoptosis, necrosis, senescence, and cell loss. CEP lesions were characterized by cartilage erosion, localized thinning, fissures, erosions or nodes, and avulsion from the BEP. ECM structure of the CEP was evaluated for cartilage thinning, structural disorganization, loss of boundary demarcation (tidemark and cement lines), fibrocartilage infiltration, and calcification. BEP cellularity was evaluated based on bone marrow composition, specifically the presence of red bone marrow (rbm) versus adipocytes indicative of fatty yellow bone marrow (ybm), as well as signs of neovascularization. BEP lesions included changes in demarcation, enthesis integrity, and erosions or nodules. Finally, the ECM of the BEP structure was assessed for the presence of erosive nodules, trabecular remodeling, changes in thickness, cartilage or fibrocartilage formation, subchondral bone sclerosis, and bone remodeling, including osteophyte formation.

**TABLE 4:** Hematoxylin and eosin (H&E) degeneration scoring system based on large animal models [64, 65, 66], focusing on cellularity, lesions, and extracellular matrix (ECM) structure of cartilaginous endplate (CEP) (a) and bony endplate (BEP) (b).

(a) Location	CEP		
Categories	Cellularity	Lesions	ECM structure
Markers	Cellular proliferation (clusters), neovascularization	Cartilage erosion, localized thinning, fissures, erosions or nodes, avulsion from BEP	Cartilage thinning, structural disorganization, boundary demarcation, fibrocartilage infiltration, and calcification.
0. Absence of degeneration	Single cells within lacunae, with no signs of necrosis, apoptosis, or senescence.	None.	Uniform thickness with a distinct boundary with BEP.
1. Early/mild degeneration	Few instances of multiple nuclei within lacunae, with possible signs of apoptosis and cell shrinkage.	Few areas of localized erosion.	Some localized thinning and disorganization.
2. Moderate degeneration	Several of multiple nuclei are present within lacunae, but without clustering, possible signs of apoptosis, necrosis, and cellular senescence, moderate cell loss, and neovascularization.	Moderate thinning, presence of fissures/nodules, and localized avulsion from the BEP.	Moderate calcification, fibrocartilage infiltration, and structural disorganization.
3. Severe degeneration	Abundant clustering of clonally derived cells, with evidence of cell loss, and possible signs of apoptotic, necrotic, and senescent cells, and extensive neovascularization.	Significant loss of thickness, large fissures, and extensive areas of avulsion from the BEP.	Extensive calcification and fibrocartilage infiltration.

(b) Location	BEP		
Categories	Cellularity	Lesions	ECM structure
Markers	Bone marrow alterations and neovascularization.	Demarcation, enthesis state, erosions or nodules, and osteophyte formation.	Erosive nodules, trabecular remodeling, changes in thickness, cartilage formation, sclerotic subchondral bone, cartilage or fibrocartilage, bone remodeling, and osteophyte formation.
0. Absence of degeneration	Hematopoietic bone marrow. red bone marrow (rbm) instead of yellow bone marrow (ybm)	A distinct boundary with the CEP.	Thin subchondral bone plate with distinct vertebral trabecular elements.
1. Early/mild degeneration	Localized areas of fibrovascular/fatty ybm at sites of CEP damage.	Scattered irregularities at the CEP interface.	Small node with rupture of the BEP. Limited areas of fibrocartilage infiltration into the subchondral bone, with or without a sclerotic margin.
2. Moderate degeneration	Moderate‐sized areas of fibrovascular and/or fatty bone marrow extending into the VB.	Moderate irregularities at the CEP boundary.	Moderately sized nodes and/or erosions, with adjacent sclerosis or fibrovascular changes in the bone marrow. Sclerosis of the subchondral bone plate.
3. Severe degeneration	Extensive fibrovascular bone marrow changes.	Erosive nodules extending into the vertebra, with a BEP compression fracture.	Extensive bony sclerosis.

Images of the full CEP section were taken using the Multi‐position Mosaic feature. The CEP ROI was then traced as mentioned above (section: “Histological Examination”), and its area was calculated, and the nucleus of each cell in the ROI was labeled using the multi‐point tool. All nuclei were labeled, including the ones showing signs of apoptotic, necrotic, and/or senescent cells, but empty lacunae were disregarded. The count was then measured and divided by the area to get the cell density of each sample.

### 3.5 Immunohistochemistry

Additional sections were prepared as described above (section: “Histological Examination”) and mounted on charged slides for immunohistochemistry (IHC). IHC was performed using 3,3‐Diaminobenzidine tetrahydrochloride (DAB) (Sigma Aldrich D5905) staining following the protocol described by Binch et al. [69]. The sections were deparaffinized, rehydrated, and endogenous peroxidase activity was blocked prior to antigen retrieval. Antigen retrieval was performed using one of three methods: none, enzyme, or heat (Table 5). For enzyme retrieval, sections were incubated in TBS containing 0.1% (w/v) CaCl_2_ dihydrate (Honeywell 223 506) and 0.01% (w/v) α‐chymotrypsin (Sigma Aldrich 1 002 472 729) for 30 min at 37°C. For heat retrieval, sections were submerged in 0.05 M Tris (Fisher T/3710/60) at pH = 9.5 preheated to 60°C, irradiated in an 800 W microwave for 5 min at 40% power, left to stand for 1 min, irradiated for another 5 min at 20% power, and then left to cool for 15 min at room temperature. Following antigen retrieval, sections were washed in TBS. Non‐specific antibody‐protein interactions were blocked using normal serum matched to the secondary antibody species for 1 h at room temperature in a humidified staining tray to prevent drying. Primary antibodies were applied overnight at 4°C. The sections were incubated with primary antibodies against anabolic matrix staining markers COL2 [70] and ACAN [71] in addition to catabolic cytokines IL‐1β and TNF, and matrix‐degrading enzymes MMP3 and ADAMTS4 [59, 60, 61, 62] (Table 5). Parallel sections were incubated with species‐matched IgG controls (rabbit, Abcam ab37415; mouse, Abcam ab18394; Goat, Abcam ab37373) at equivalent concentrations. The following day, sections were washed in TBS and incubated with secondary antibodies (Table 5) for 30 min. After further TBS washes, ABC Elite reagent (Vector Laboratories PK‐7100) was applied for 30 min. Sections were washed again in TBS, and DAB solution (0.08% H_2_O_2_ with 0.65 mg/mL DAB in TBS) was applied for 20 min. Sections were then washed under running cold tap water for 5 min, counterstained with Mayer's hematoxylin (Leica Biosystems  3801582E) for 1 min, and blued under running tap water for another 5 min. Finally, sections were dehydrated in ethanol, cleared in Xylene (Sigma Aldrich 214 736), and mounted with coverslips using Pertex (Leica Biosystems 3808707E). Each TBS wash was repeated 3 times for 5 min, and the primary and secondary antibodies were purchased from Abcam, Cambridge, UK, or Invitrogen (Table 5). Slides were scanned at 20× magnification using a slidescanner (PANNORAMIC 250 Flash II DX, 3DHistech). Quantification of immunostaining was performed using a QuPath [72]‐based semi‐automatic pipeline [73] optimized for low‐cellularity tissues. To enable assessment of the CEP response and potential cross‐talk with adjacent tissues, regions of interest were defined for the NP/AF, CEP, and BEP of each sample. The stain vector was automatically estimated, cells were automatically detected, and the classifier trained using 15 representative regions to distinguish positive cells, negative cells, and false detections. Samples containing fewer than 150 cells were excluded to prevent statistical bias, and immunopositivity was expressed as the percentage of positive cells for each antigen and tissue region.

**TABLE 5:** Primary and secondary antibodies used for immunohistochemical (IHC) staining with their clonality, dilution, and antigen retrieval methods.

Target antibody	Clonality	Dilution	Antigen retrieval	Secondary antibody	Dilution
MMP3 (ab53015)	Rabbit polyclonal	1:400	Enzyme	Goat anti rabbit (ab6720)	1:500
Aggrecan (ab3778)	Mouse monoclonal	1:200	Heat	Rabbit anti mouse (ab6727)	1:500
Collagen Type II (ab3092)	Mouse monoclonal	1:100	Enzyme	Rabbit anti mouse (ab6727)	1:500
ADAMTS4 (ab185722)	Rabbit polyclonal	1:200	None	Goat anti rabbit (ab6720)	1:500
IL‐1β (Invitrogen M421B)	Mouse Monoclonal	1:100	Heat	Rabbit anti mouse (ab6727)	1:500
TNF (ab6671)	Rabbit polyclonal	1:200	Heat	Goat anti rabbit (ab6720)	1:500

### 3.6 Statistical Analysis

GraphPad Prism version 9.0.0 for Windows (GraphPad Software, Boston, Massachusetts, USA) was used to conduct normality tests and statistical analyses. A normality test was performed to determine the type of statistical tests that could be conducted on the results of each downstream analysis. GAG/DNA passed both the Shapiro–Wilk and the Kolmogorov–Smirnov normality test, and, therefore, parametric significance tests were used to assess the effects of the environments on the samples. GAG/DNA in the tissues had only one variable, which was the environment, so a one‐way ANOVA was performed, while GAG/DNA released in the media had an additional variable, time, so a two‐way ANOVA test was performed. After the ANOVA test, Tukey's multiple comparisons test was performed to check which environment exactly differs from the rest. Due to the bounded nature of the degeneration scores (0–18) and IHC percentage data (0%–100%), the non‐parametric Kruskal–Wallis test was performed, followed by Dunn's multiple comparisons test.

## 4 Results

### 4.1 In Silico Modeling Predicts Cytokine Enhancement of Degeneration

Simulating degenerative conditions in the absence of cytokines predicted a negligible transition to a degenerative phenotype, with fewer than 1% of chondrocytes affected (2/1100). In contrast, inclusion of cytokines resulted in a complete shift, with 100% of chondrocytes exhibiting a degenerative phenotype. At the protein level within the *in silico* model (Supplementary Figure S.1a), 25 out of 74 components were significantly different from healthy controls under degenerative conditions without cytokines, whereas 55 differed from degenerated controls. With cytokines, this pattern was reversed, with 55 components differing from healthy controls and only 1 from degenerated controls. A similar trend was observed at the gene level within the *in silico* model (Supplementary Figure S.1b). Without cytokines, 21 components differed significantly from healthy controls and 46 from degenerated controls. While with cytokines, 47 components differed from healthy controls, and only 1 from degenerated controls. When assessed globally (Figure 3 and Supplementary Figure S.1c), degenerative conditions without cytokines resulted in 35 components differing from healthy controls and 73 from degenerated controls. In contrast, with cytokines, all 74 components differed from healthy controls and none from degenerated controls.

**FIGURE 3:**
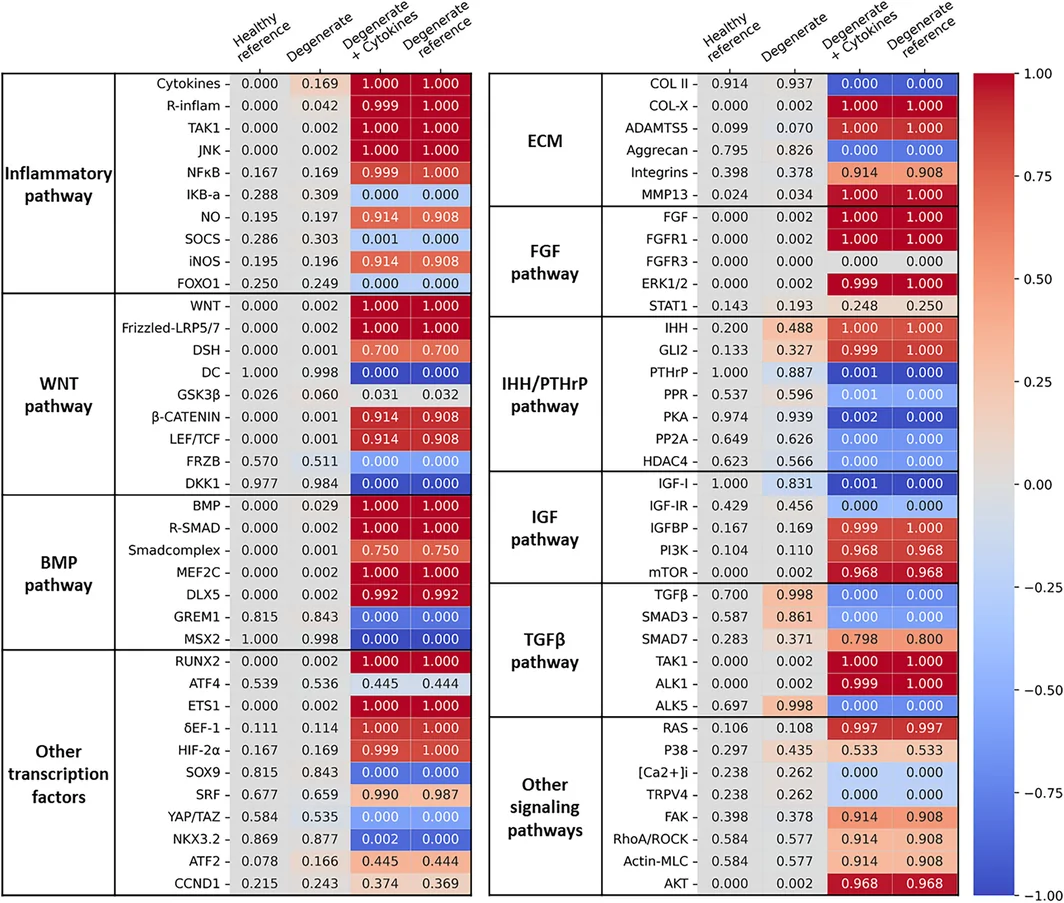
Normalized global activity of all the monitored components as predicted by the in silico model for the reference healthy chondrocytes (healthy control), chondrocytes in degenerative conditions without cytokines, chondrocytes in degenerative conditions with cytokines, and the reference degenerated chondrocytes (degenerated control). Controls have been defined in previous studies [36, 37]. The heatmap shows the deviation (in absolute values) of each component from the reference healthy environment.

## 5 Downstream Analysis

### 5.1 CT Scanning Showed Structural Stability After 14 Days of Culture Within Ex Vivo Models

CT scans revealed that the primary difference among the three bovine subjects was the absence of a visible growth plate in the VB of bovine 2. In contrast, all samples from bovines 1 and 3 exhibited a clearly identifiable growth plate, located consistently within approximately 2 mm from the BEP, with average distances of 2.32 mm for bovine 1 and 1.82 mm for bovine 3. Although some samples from bovine 2 had bone heights exceeding 11 mm, no visible growth plate was detected. This suggests that bovine 2 may have been more skeletally mature. However, structural features of the growth plate region were still distinguishable in these samples. These features were located at a similar position relative to the BEP as in bovines 1 and 3, with an estimated closed growth plate distance of 2.05 mm in bovine 2, based on anatomical landmarks (Supplementary Figure S.2). One sample of bovine 2 of the degenerate + IL‐1β condition had multiple Schmorl's nodes where the BEP‐CEP boundary was not well defined. That sample had extreme thinning of the cartilage, and so did one sample of the same bovine of the degenerate + TNF condition, although that one did not show Schmorl's nodes, so these two samples were discarded from the analysis as the features represented donor variation rather than effects of culture conditions. All other samples showed a clear distinction between the bone, cartilage, and soft tissues. Aside from the STB, the thickness of each tissue was comparable between the samples without statistical significance, regardless of the environment (*p* > 0.05) (Figure 4). One sample of the same bovine in the degenerate + IL‐1β condition did show a small section with unusual calcification, but still had clear tissue boundaries. The thickness of each tissue for all the samples were on average, 3.9 mm for the soft tissues, 0.345 mm for the ncCEP, 0.304 mm for the cCEP, 0.701 mm for the BEP, and 2.072 mm for the STB.

**FIGURE 4:**
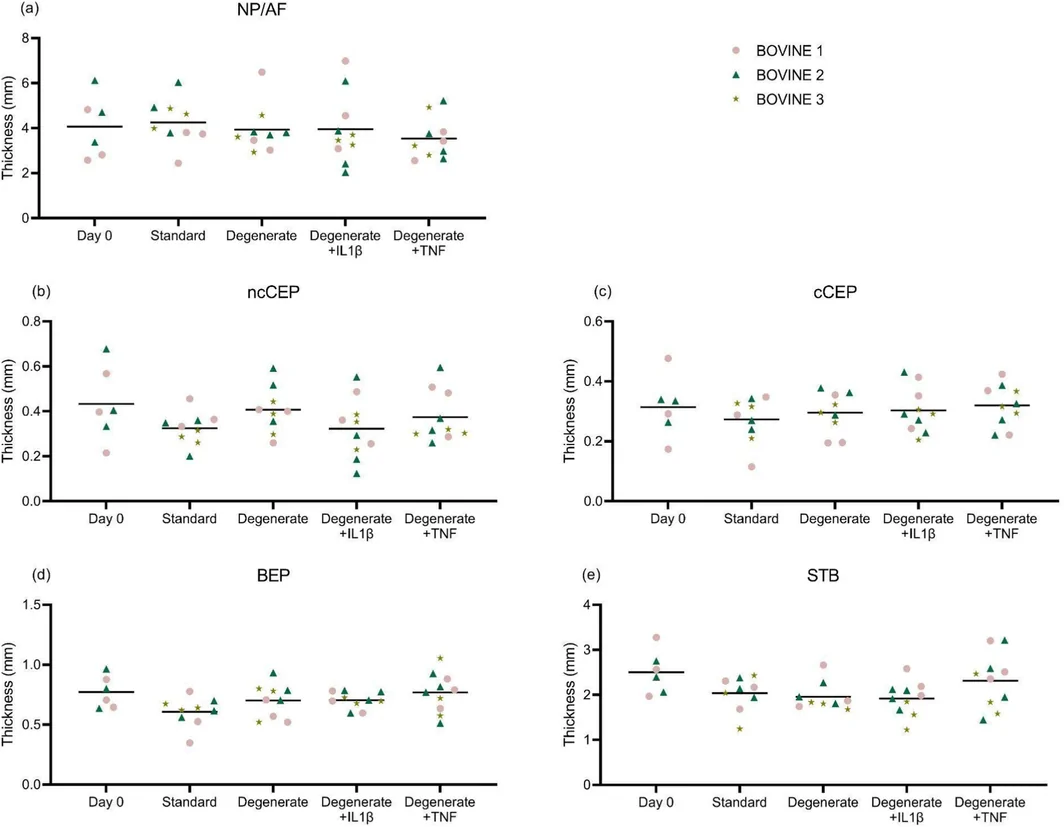
Thickness of (a) nucleus pulposus and/or annulus fibrosus (NP/AF), (b) non‐calcified cartilaginous endplate (ncCEP), (c) calcified cartilaginous endplate (cCEP), (d) bony endplate (BEP), and (e) subchondral trabecular bone (STB), in the explants as measured in the μCT scans using 3D Slicer. Standard, degenerate, degenerate + IL‐1β, and degenerate + TNF media are all measured at day 14.

### 5.2 GAG/DNA Did Not Change in the Tissues During Culture, but TNF Induced GAG Release to the Medium

On average, all three bovine cultures showed comparable GAG/DNA levels in the tissue on day 0, and all cultured samples for 14 days in standard medium, and degenerate groups with no significant differences in GAG/DNA seen between bovine donors (Figure 5a).

**FIGURE 5:**
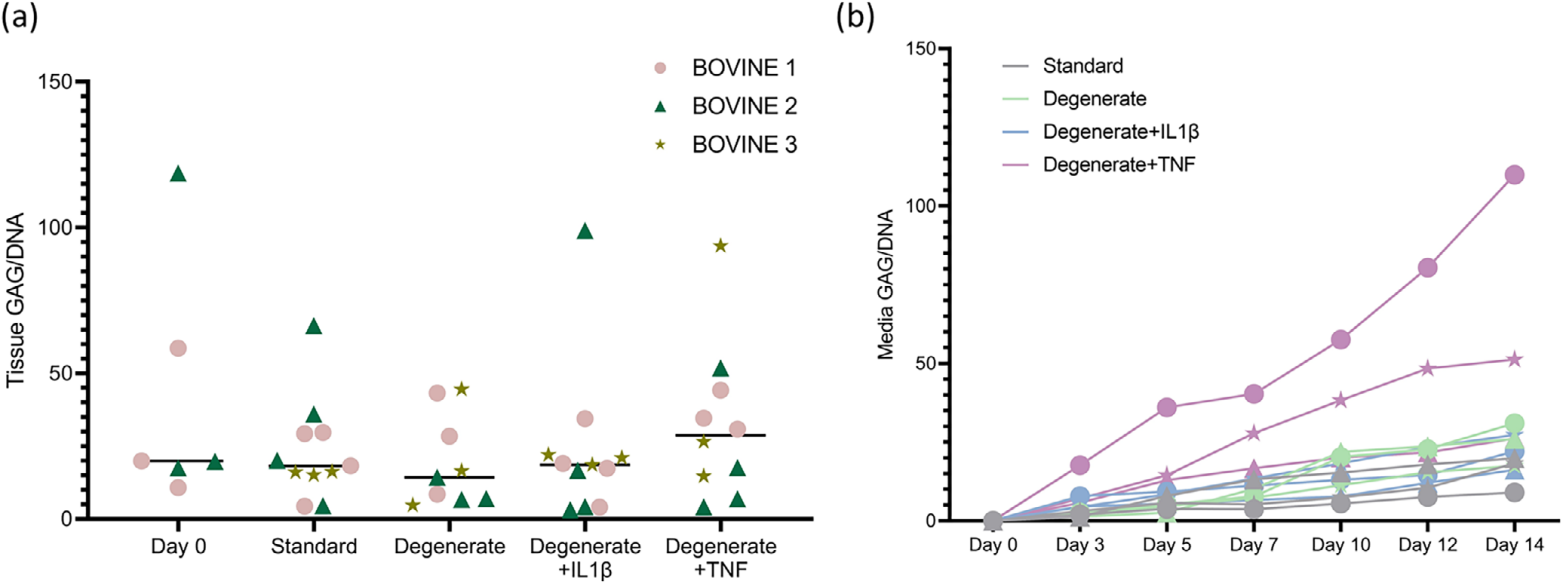
Quantification of (a) GAG/DNA in the tissues of the digested explants for the three bovines in each environment where standard, degenerate, degenerate + IL‐1β, and degenerate + TNF media are all measured at day 14, and (b) cumulative GAG/DNA released in each medium by all three bovine explant samples; note media samples for equivalent treatment groups were pooled at each time point for analysis. Statistical analysis was performed and significant effects of environment and time were observed for cumulative GAG/DNA release (b), where the GAG/DNA in the degenerate + TNF media were significantly greater compared to the standard group at days 10, 12, and 14.

There were clear significant effects of both environment and time on the cumulative GAG/DNA secretion (*p* < 0.001 for both environment and time) (Figure 5b). Post hoc analysis, using Tukey's multiple comparisons test, showed that at days 3, 5, and 7, none of the pairwise comparisons reached statistical significance (*p* > 0.05). From day 10 onward, the degenerate + TNF condition exhibited significantly higher cumulative GAG/DNA release compared to standard conditions (*p* = 0.009, *p* = 0.001, and *p* < 0.001 for days 10, 12, and 14, respectively) while the degenerate and degenerate + IL‐1β differences remained insignificant compared to standard conditions at all measured days.

### 5.3 Histological Examination Showed Decreased Matrix Staining When Cultured in Cytokine‐Simulated Media

Macroscopically, AB, SafO, and MTC staining were more intense in the samples of day 0 conditions, followed by standard, then degenerative samples. The distinction between the CEP and surrounding soft tissues was more evident under day 0 conditions, with the soft tissues exhibiting stronger AB staining than the CEP, while the CEP exhibited stronger SafO staining compared to the NP/AF regions. By day 14, this contrast was reduced. While still observable in the standard medium samples, it was notably diminished in the degenerative conditions (Figures 6a, 7a, and 8a).

**FIGURE 6:**
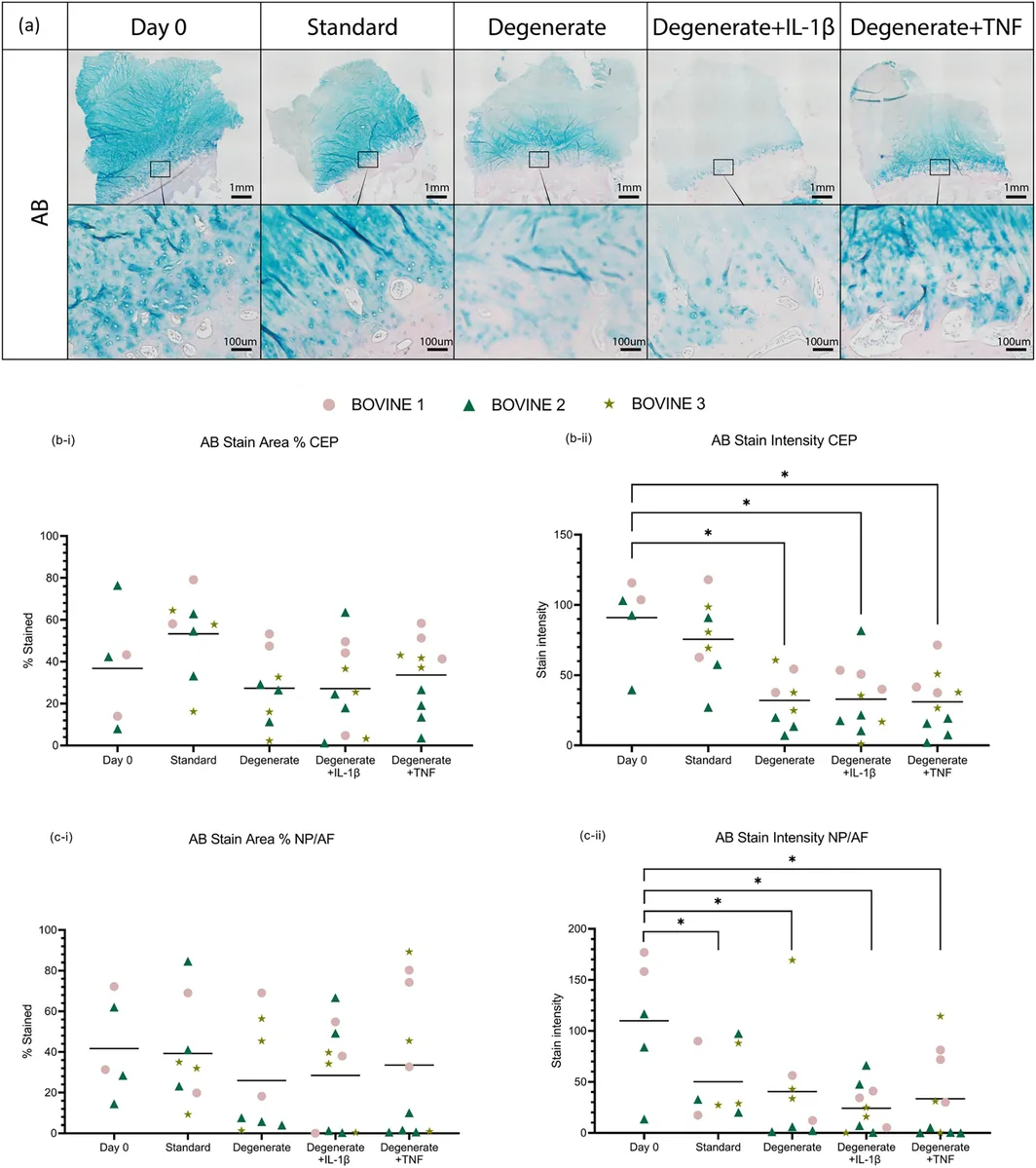
Alcian blue (AB) stain (a) representative samples with (i) the quantified percentage stained area and (ii) staining intensity in the (b) cartilaginous endplate (CEP) and the (c) nucleus pulposus and/or annulus fibrosus (NP/AF) between the five different media conditions. The box shows the location of the magnified region below each figure. Scale bar = 1 mm for the figures and 100 μm for the magnified regions. Standard, degenerate, degenerate + IL‐1β, and degenerate + TNF media are all measured at day 14. * = *p* < 0.05.

**FIGURE 7:**
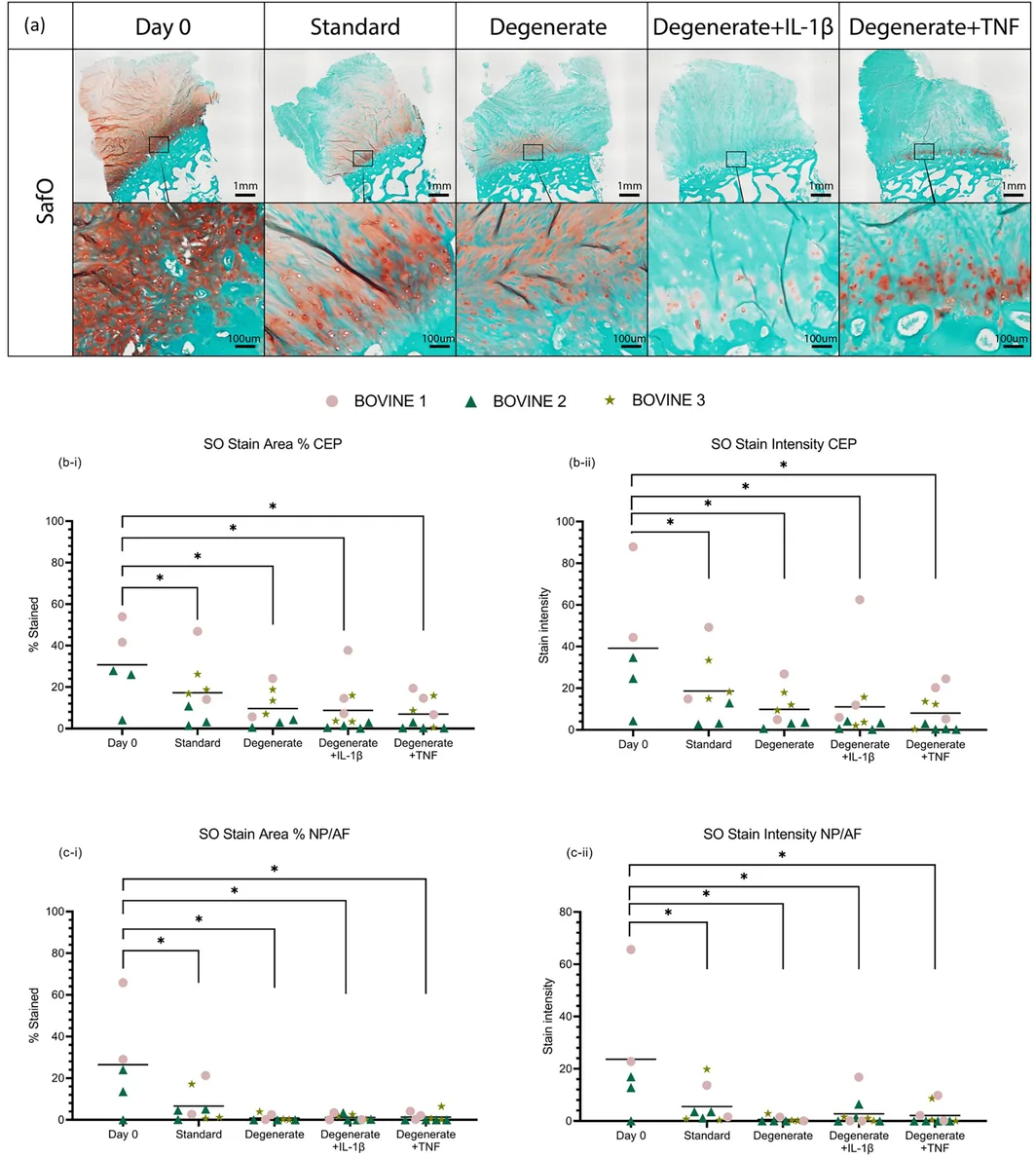
Safranin O (SafO) stain (a) representative samples with (i) the quantified percentage stained area and (ii) staining intensity in the (b) cartilaginous endplate (CEP) and the (c) nucleus pulposus and/or annulus fibrosus (NP/AF) between the five different media conditions. The box shows the location of the magnified region below each figure. Scale bar = 1 mm for the figures and 100 μm for the magnified regions. Standard, degenerate, degenerate + IL‐1β, and degenerate + TNF media are all measured at day 14. * = *p* < 0.05.

**FIGURE 8:**
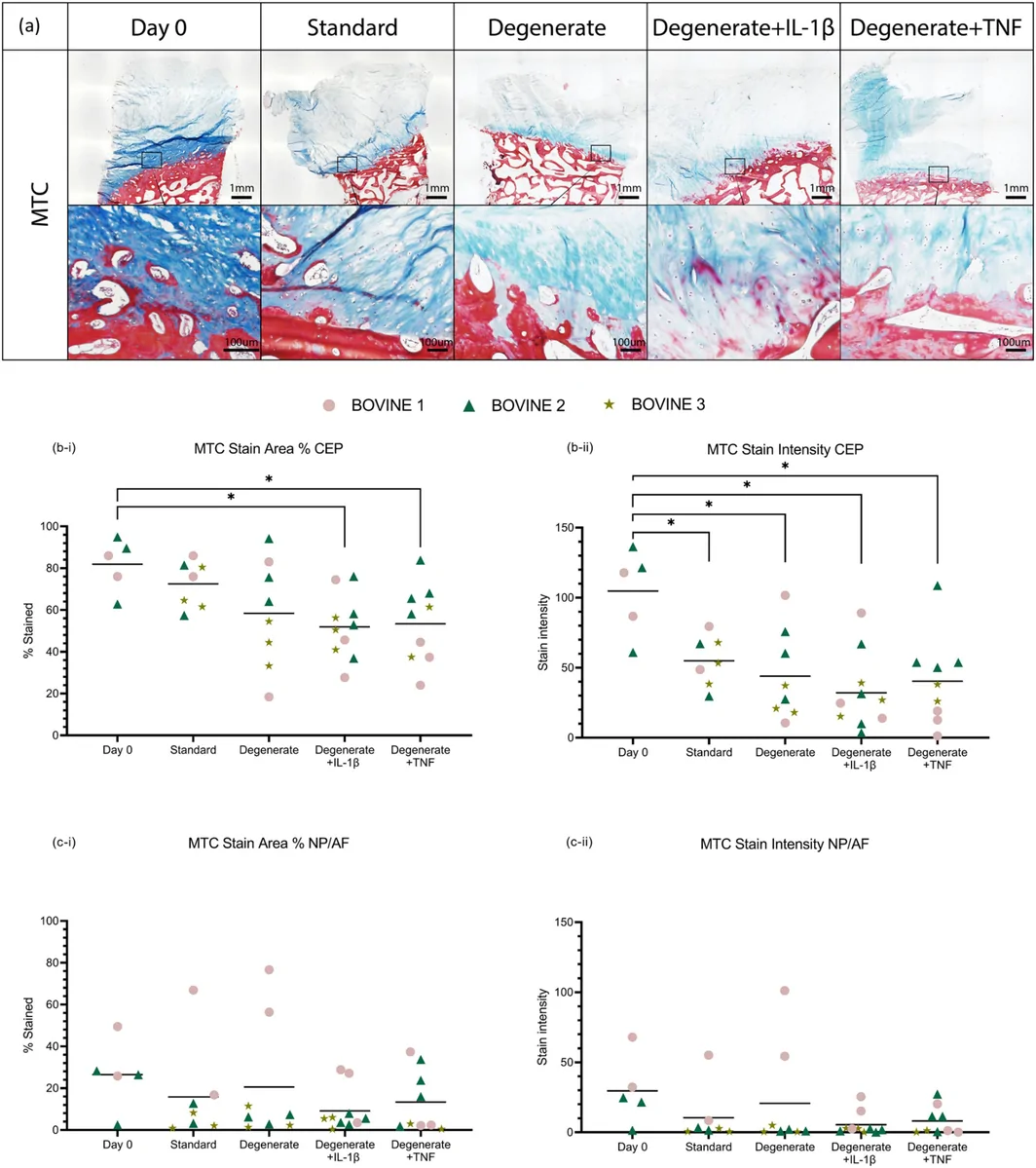
Masson Trichrome (MTC) stain (a) representative samples with (i) the quantified percentage stained area and (ii) staining intensity in the (b) cartilaginous endplate (CEP) and the (c) nucleus pulposus and/or annulus fibrosus (NP/AF) between the 5 different media conditions. The box shows the location of the magnified region below each figure. Scale bar = 1 mm for the figures and 100 μm for the magnified regions. Standard, degenerate, degenerate + IL‐1β, and degenerate + TNF media are all measured at day 14. * = *p* < 0.05.

The staining area and the staining intensity were quantified for both the CEP and the soft tissues (NP/AF). Concerning the CEP, quantification of the stained area for AB showed similar percentages across all conditions (*p* > 0.05) (Figure 6bi). However, staining intensity significantly decreased in the three degenerate media treatment groups, particularly in the degenerate + TNF medium, while remaining comparable between the day 0 condition and the standard medium following 14 days (*p* = 0.414 for standard, *p* < 0.001 for degenerate, *p* < 0.001 for degenerate + IL‐1β, and *p* < 0.001 for degenerate + TNF compared to day 0) (Figure 6bii). Concerning the NP/AF tissues, the stained area for AB also showed similar percentages across all conditions (*p* > 0.05) (Figure 6ci) but the staining intensity dropped significantly in all conditions compared to day 0 (*p* = 0.03 for standard, *p* = 0.001 for degenerate, *p* = 0.001 for degenerate + IL‐1β, and *p* = 0.003 for degenerate + TNF compared to day 0) (Figure 6cii).

In contrast, SafO exhibited a significant reduction in both stained area (Figure 7bi) and intensity (Figure 7bii) across all media compared to the day 0 condition, with the most pronounced decreases observed in the three degenerate media conditions, especially the degenerate + IL‐1β and degenerate + TNF (for stain area percentage *p* = 0.019 for standard, *p* < 0.001 for degenerate, *p* < 0.001 for degenerate + IL‐1β, and *p* < 0.001 for degenerate + TNF compared to day 0 while for stain intensity *p* = 0.028 for standard, *p* = 0.001 for degenerate, *p* = 0.001 for degenerate + IL‐1β, and *p* < 0.001 for degenerate + TNF compared to day 0). Concerning the NP/AF tissues, SafO exhibited a significant reduction in both stained area (Figure 7ci) and intensity (Figure 7cii) across all media compared to the day 0 (for stain area percentage *p* = 0.001 for standard, *p* < 0.001 for degenerate, *p* < 0.001 for degenerate + IL‐1β, and *p* < 0.001 for degenerate + TNF compared to day 0 while for stain intensity *p* = 0.004 for standard, *p* < 0.001 for degenerate, *p* < 0.001 for degenerate + IL‐1β, and *p* < 0.001 for degenerate + TNF compared to day 0).

MTC showed consistent stained area percentages across the day 0 conditions, standard, and degenerate media, with higher values in the cytokine conditions (*p* < 0.05 for standard, *p* < 0.05 for degenerate, *p* = 0.025 for degenerate + IL‐1β, and *p* = 0.031 for degenerate + TNF compared to day 0) (Figure 8bi). Yet, MTC staining intensity significantly declined in all media, most notably in the degenerate + IL‐1β condition (*p* = 0.05 for standard, *p* = 0.007 for degenerate, *p* < 0.001 for degenerate + IL‐1β, and *p* = 0.002 for degenerate + TNF compared to day 0) (Figure 8bii). Of the degenerate media, degenerate + IL‐1β showed the least AB intensity, while degenerate + TNF showed the least MTC intensity, and both degenerate + IL‐1β and degenerate + TNF equally showed the lowest intensity in SafO compared to the degenerate medium without cytokines. Concerning the NP/AF tissues, MTC showed consistent stained area percentages (Figure 8ci) and staining intensity (Figure 8cii) across all conditions (*p* < 0.05 compared to day 0).

There was no clear division between the ncCEP and the NP in AB and H&E due to their similar biochemical composition. Therefore, MTC staining was used to differentiate between the CEP and the NP since the intensity of the blue stain was relative to the collagen content, where the NP was pale blue, almost white, while the CEP showed dark staining; however, staining intensity of the CEP was indistinguishable from the AF staining intensity (Figure 9).

**FIGURE 9:**
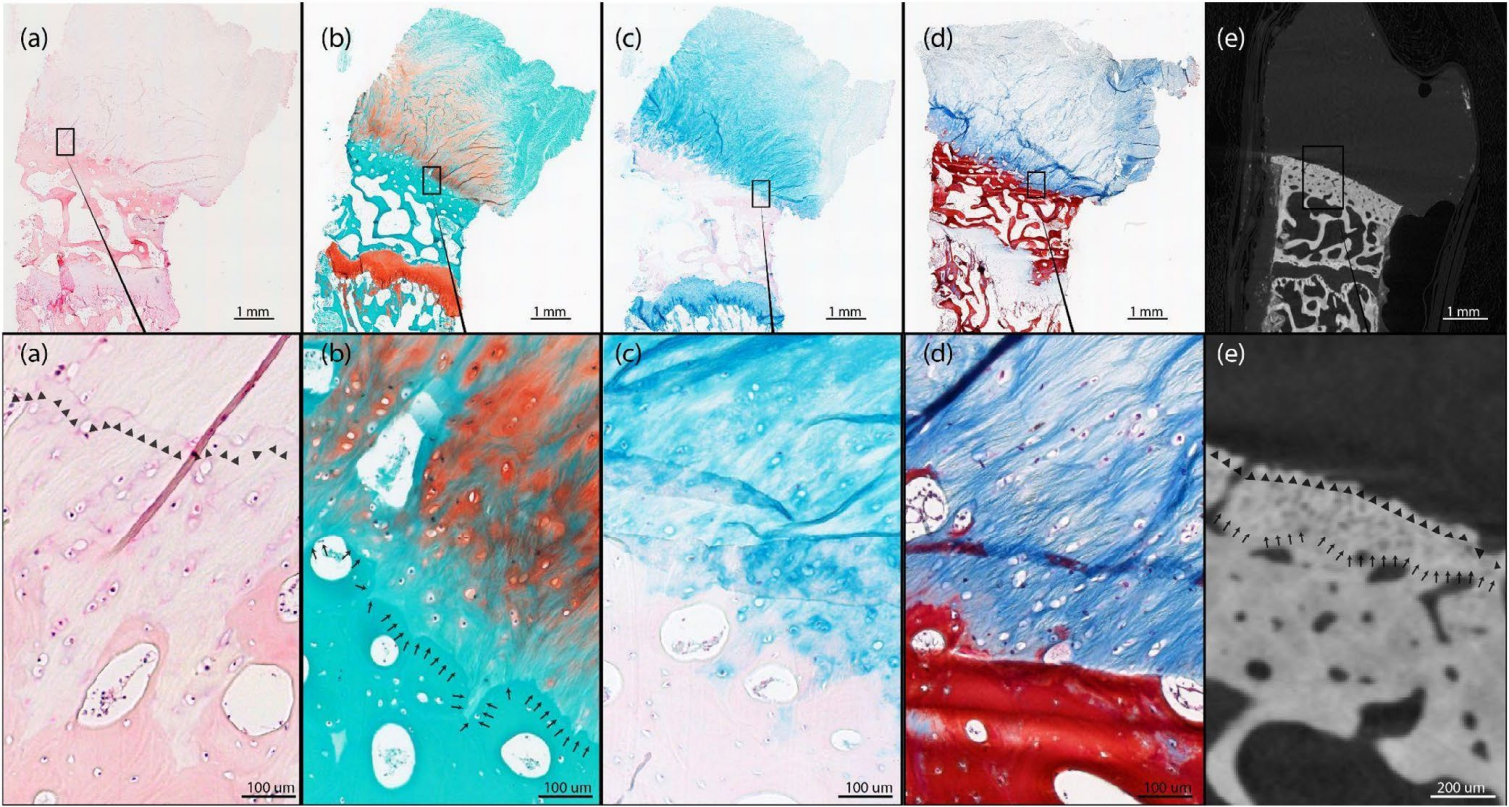
Histological staining of (a) hematoxylin and eosin (H&E), (b) safranin O (SafO), (c) alcian blue (AB), and (d) Masson's trichrome (MTC) in addition to (e) the μCT scan counterpart, where ▲ traces the tidemark and ↑ represents the cement line. This is a sample from the standard condition.

All the samples showed a well‐organized subchondral bone with a clear cement line that divided the cartilaginous part and the osteogenic part of the sample. Tidemarks were visible in most of the explant samples, especially in H&E staining, where they appeared as a thin, wavy purple line (Figure 9a). That was clearly shown in the SafO staining, where the bone was dark blue while the cCEP was pale bluish‐pink (Figure 9b).

All the samples showed uniformly distributed scattered BEP osteocytes and dense CEP chondrocytes, but only the day 0 and standard medium samples had uniform organization of CEP chondrocytes, while the degenerative samples showed more clustering of these cells (Figure 10).

**FIGURE 10:**
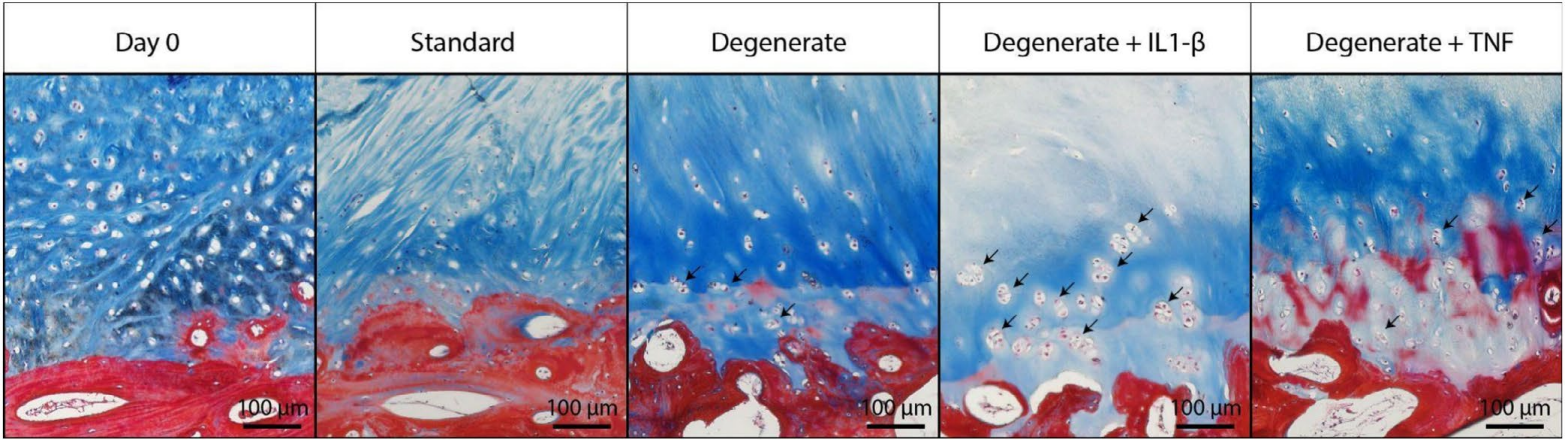
Masson's trichrome (MTC) staining showing uniformly distributed chondrocytes in day 0 and standard medium samples versus clustered chondrocytes in samples in degenerative media with and without cytokines. The arrows indicate the clustering of 3 or more chondrocytes.

They all showed an abundance of chondrocytes and osteocytes in their respective regions. Osteoclasts and osteoblasts were present in a very small number, but appeared to be more in the degenerative samples than the day 0 conditions and standard medium samples, although this was not quantified.

Degeneration scoring of the vertebral endplate (Figure 11) revealed a clear and progressive increase across experimental conditions. Statistically significant differences were observed between all sequential groups, with scores increasing from day 0 to standard culture, from standard to degenerate conditions, and further from degenerate to cytokine‐treated groups (all *p* < 0.001) (Figure 12).

**FIGURE 11:**
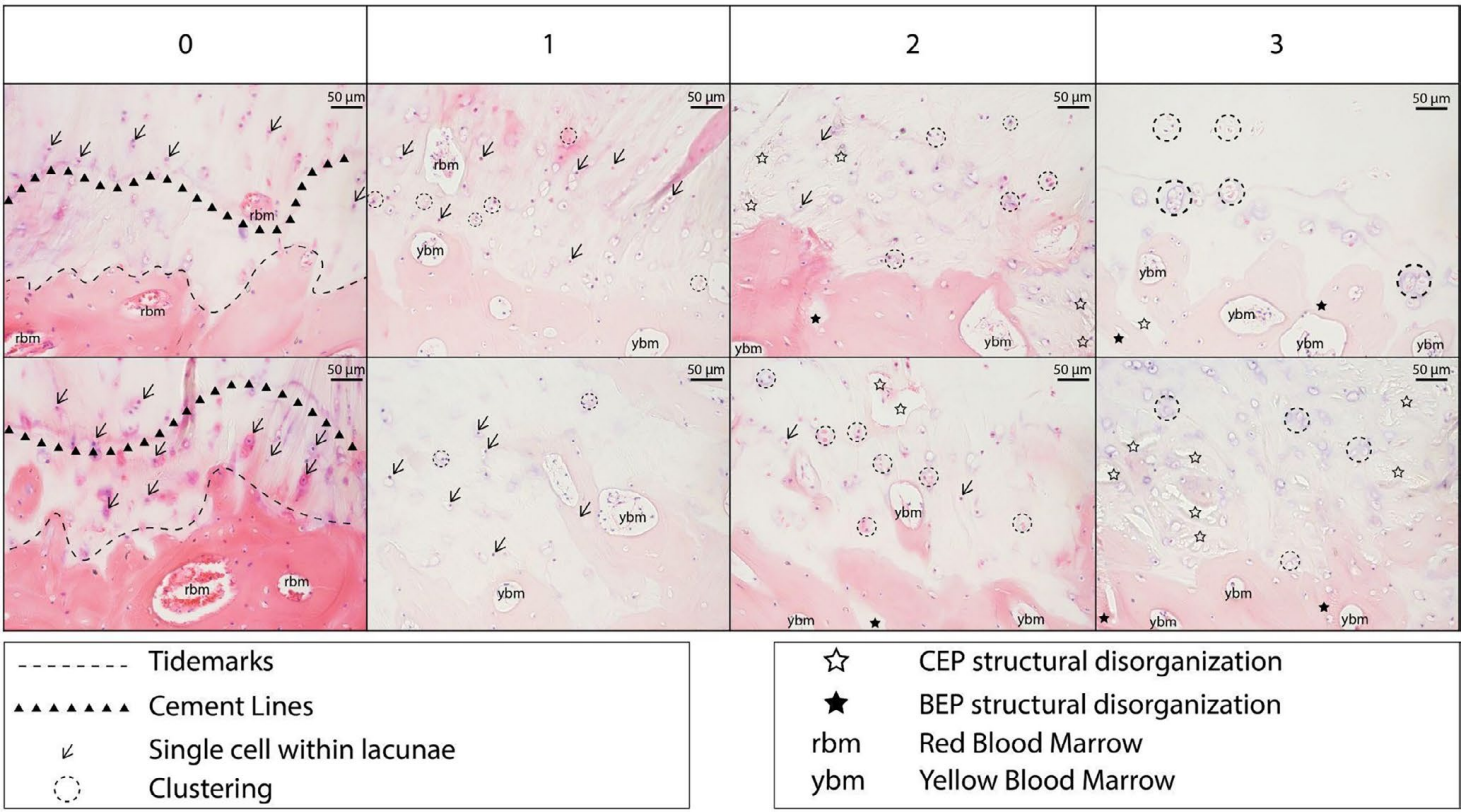
Two representative examples (rows) of the hematoxylin and eosin (H&E) stained cartilaginous endplate (CEP) and bony endplate (BEP) sections by degeneration score from 0 to 3 (columns) based on the system presented in Table 4. Scale bar = 50 μm.

**FIGURE 12:**
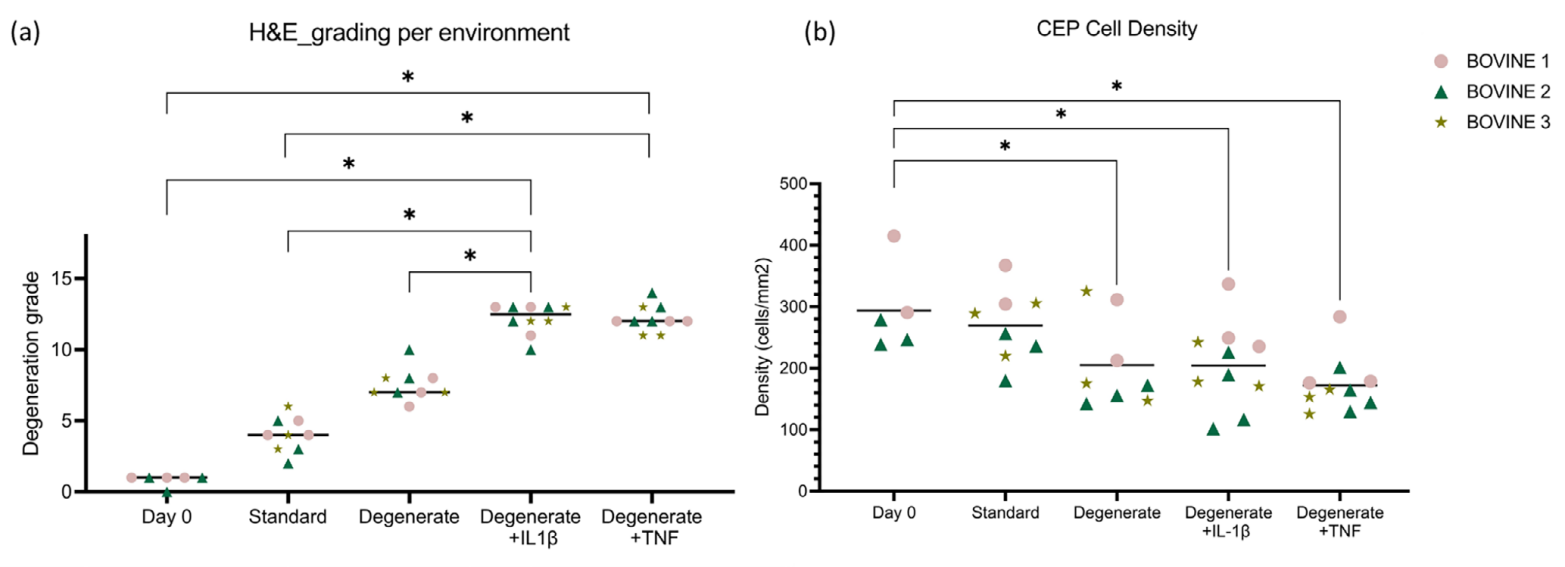
(a) Degeneration scoring grades for the three bovines in their different environments. (b) The chondrocyte cell density of the cartilaginous endplate (CEP) in the three bovines in their different environments. Standard, degenerate, degenerate + IL‐1β, and degenerate + TNF media are all measured at day 14. * = *p* < 0.05.

Specifically, histological features of degeneration in both cytokine‐induced conditions (degenerate + IL‐1β, and degenerate + TNF) were significantly higher than day 0 and standard conditions (*p* < 0.001) (Figure 12a). Histological features of degeneration in the degenerate + IL‐1β were also significantly higher than the degenerate condition without cytokines (*p* < 0.05), while degenerate + TNF did not show a significant difference compared to the degenerate condition without cytokines (Figure 12a). No significant difference was observed between the degenerate + IL‐1β and degenerate + TNF groups (*p* > 0.05) (Figure 12a), indicating comparable effects of both cytokines on endplate degeneration.

The day 0 chondrocyte cell density in the CEP averaged approximately 300 cells/mm^2^. Relative to day 0, a significant reduction in cell density was observed in degenerate (*p* = 0.014), degenerate + IL‐1β (*p* = 0.007), and degenerate + TNF (*p* < 0.001) conditions (Figure 12b), while no significant difference was found between day 0 and standard (*p* > 0.05) (Figure 12b). Similarly, compared to standard conditions, cell density was significantly reduced in degenerate (*p* = 0.038), degenerate + IL‐1β (*p* = 0.020), and degenerate + TNF (*p* < 0.001) (Figure 12b).

No significant differences were detected among degenerate, degenerate + IL‐1β, and degenerate + TNF groups (all *p* > 0.05) (Figure 12b), indicating that although degenerative conditions and cytokine exposure reduced cell density relative to baseline and standard culture, their effects were not significantly different from each other.

### 5.4 Immunohistochemistry Showed Responses in CEP and NP/AF Tissues but Not in BEP

Immunohistochemical analysis (Figure 13) revealed distinct spatial patterns across anabolic, catabolic, and matrix‐degrading markers. Anabolic markers were primarily localized to the CEP, with ACAN showing the highest immunopositivity in the CEP, moderate staining in the NP and AF, and low levels in the BEP, while COL2 was strongly expressed in all regions, with the highest levels in the BEP. Catabolic markers were predominantly detected in the NP/AF and CEP, with both IL‐1β and TNF showing strong immunopositivity in these regions and comparatively lower expression in the BEP. In contrast, matrix‐degrading enzymes were more prominent in the BEP and NP/AF, as ADAMTS4 expression was high in these regions but low in the CEP, and MMP3 followed a similar trend, with the highest immunopositivity in the BEP, moderate levels in the NP/AF, and low expression in the CEP.

**FIGURE 13:**
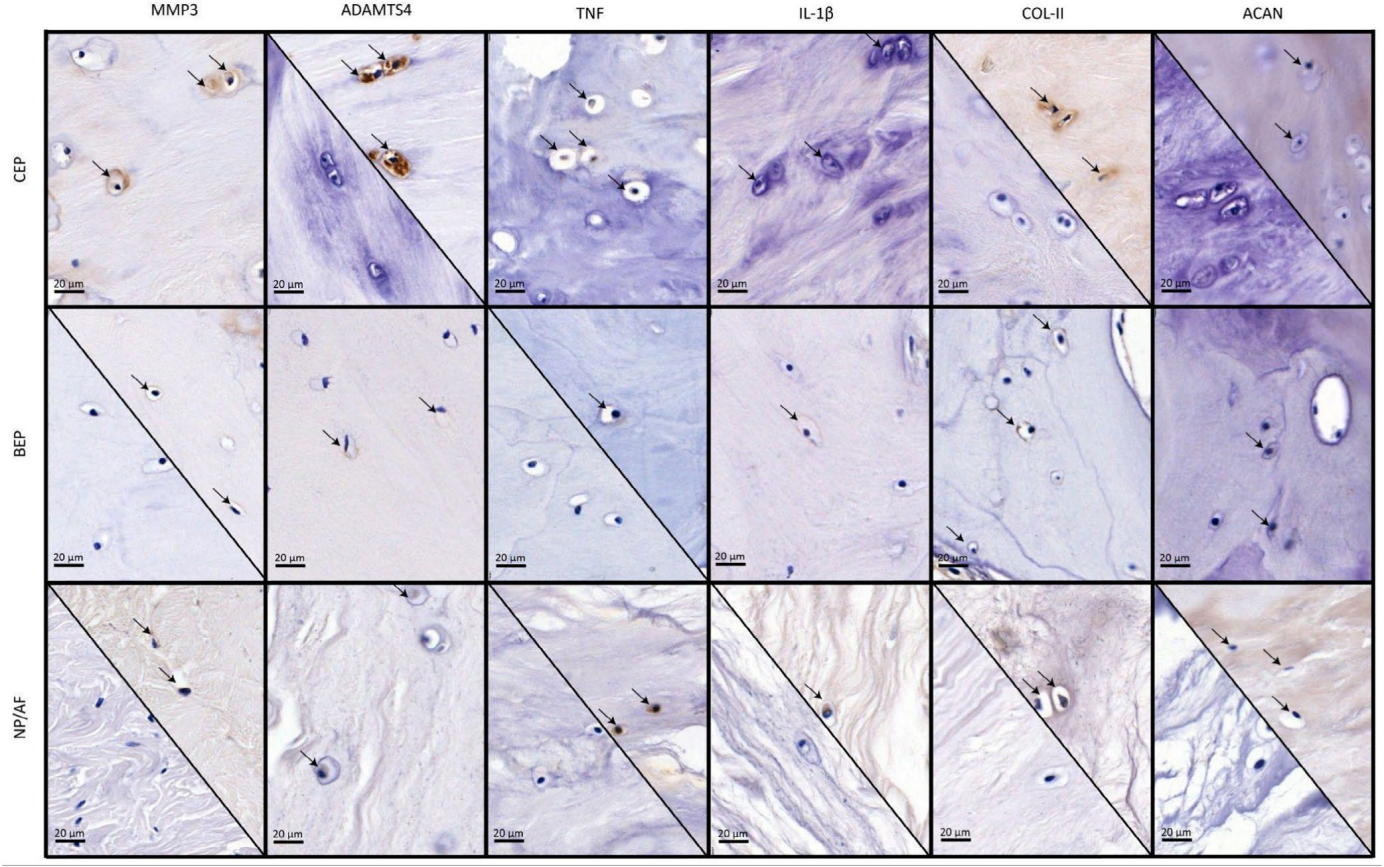
Immunohistochemical cell positivity and negativity of anabolic markers ACAN and COL2, catabolic markers IL‐1β and TNF, and matrix‐degrading markers ADAMTS4 and MMP3 in the cartilaginous endplate (CEP), bony endplate (BEP), and nucleus pulposus and/or annulus fibrosus (NP/AF). The black arrows (→) show the positively stained cells in each tissue for each antibody. When positive and negative cells were not present within the same field of view, representative regions were shown separately within the same panel and divided by a diagonal line, with the positive results shown in the bottom triangle. Scale bars = 20 μm.

Cytokine stimulation produced highly tissue‐dependent effects across anabolic and catabolic markers (Figure 14). In the CEP, regarding anabolic markers, ACAN remained the most abundant marker, showing a decreasing trend following culture, but did not reach significance between conditions (*p* > 0.05) (Figure 14a‐i). For COL2, there was an increasing trend following culture where immunostaining was significantly higher in the standard conditions (*p* = 0.013) and the degenerate + IL‐1β conditions (*p* = 0.0045) compared with day 0, and was significantly higher in the degenerate + IL‐1β conditions compared with the degenerate conditions without cytokines (*p* = 0.02) (Figure 14a‐ii). Among catabolic cytokines, IL‐1β immunopositivity showed a decreasing trend in all conditions compared to day 0, especially in degenerate + IL‐1β conditions (*p* = 0.04), but failed to reach significance in other conditions (Figure 14b‐i). For TNF, there was a decreasing trend in immunostaining for all conditions except degenerate + TNF conditions compared to day 0, but they failed to reach significance (Figure 14b‐ii). For matrix‐degrading enzymes, ADAMTS4 immunostaining was significantly lower in all cytokine conditions (degenerate + IL‐1β and degenerate + TNF) compared to day 0 (*p* = 0.002 with both conditions) (Figure 14c‐i). MMP3 showed an increasing trend under degenerative conditions with and without cytokines compared to day 0, but failed to reach significance (Figure 14c‐ii).

**FIGURE 14:**
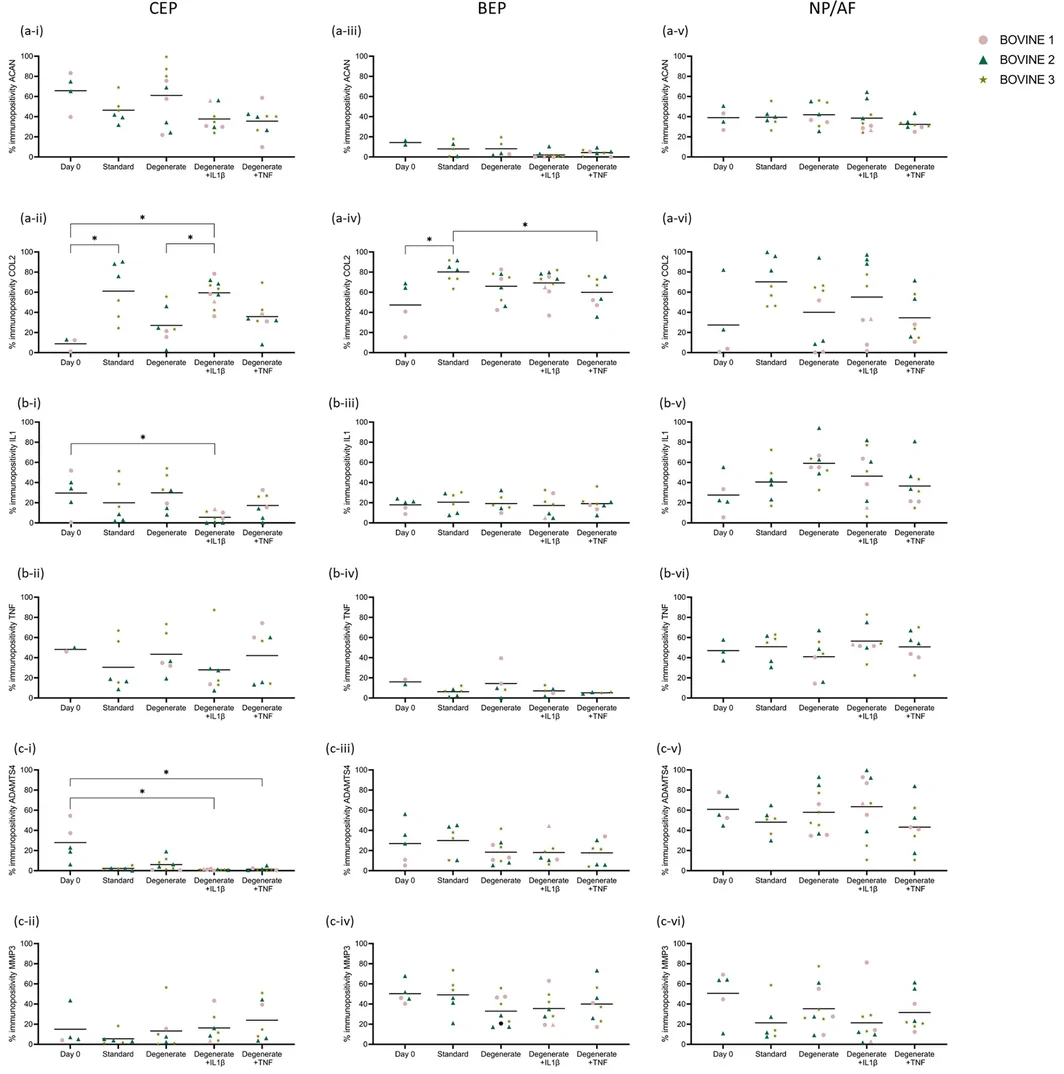
Immunopositivity of (a) anabolic markers ACAN and COL2, (b) catabolic markers IL‐1β and TNF, and (c) matrix‐degrading markers ADAMTS4 and MMP3 in the cartilaginous endplate (CEP) in the first column, bony endplate (BEP) in the second column, and nucleus pulposus and/or annulus fibrosus (NP/AF) in the third column. * = *p* < 0.05.

In the BEP, regarding anabolic markers, ACAN decreased overall compared to day 0 but did not reach significance (Figure 14a‐iii). In contrast, COL2 immunostaining increased compared to day 0, where it was significant compared to standard conditions (*p* = 0.02). There was also a significant decrease in the Degenerate +TNF condition compared to the standard condition (Figure 14a‐iv). Regarding catabolic cytokines, no significant differences in IL‐1β or TNF immunostaining were detected between the experimental conditions (*p* > 0.05 for all comparisons) (Figure 14b‐iii,iv, respectively). Similarly, for matrix‐degrading enzymes, no significant differences in ADAMTS4 or MMP3 immunostaining (Figure 14c‐iii,iv, respectively), although MMP3 showed a decreasing trend in all conditions compared to day 0.

In the NP/AF tissues, regarding anabolic markers, no significant differences were observed for ACAN or COL2 immunostaining between the experimental conditions (*p* > 0.05 for all comparisons) (Figure 14a‐v). However, COL2 did show an increasing trend among all conditions compared to day 0 (Figure 14a‐vi). Similarly, regarding catabolic cytokines, no significant differences in IL‐1β or TNF immunostaining were detected between the experimental conditions (*p* > 0.05 for all comparisons) (Figure 14b‐v,vi, respectively), but there was an increasing trend in IL‐1β in all conditions compared to day 0. Similarly, for matrix‐degrading enzymes, no significant differences in ADAMTS4 or MMP3 immunostaining were observed between any experimental conditions (*p* > 0.05 for all comparisons) (Figure 14c‐v,vi, respectively), but there was a decreasing trend in MMP3 across all cultured conditions relative to day 0.

## 6 Discussion

In this study, we combined computational modeling with a novel multi‐tissue ex vivo explant system to investigate the drivers of early EP degeneration. While the two approaches probe partially distinct perturbations, both indicated that physicochemical degenerative cues alone produced limited degeneration‐associated changes, whereas cytokine stimulation enhanced and broadened the degenerative response. *In silico* simulations predicted that chondrocytes remained stable under degenerative environmental conditions, whereas the addition of cytokines led to a complete transition toward a degenerative phenotype. This prediction was corroborated experimentally, where intact bovine IVD–VB explants exposed to IL‐1β or TNF exhibited marked proteoglycan loss and reduced cellularity, while degenerative conditions alone resulted in minimal changes. These findings support cytokines as important amplifiers of early EP degeneration under the tested conditions.

Distinct cytokine‐specific responses were observed, indicating differential roles in degeneration. TNF induced rapid and pronounced matrix degradation, as evidenced by increased GAG release and substantial reductions in proteoglycan staining. In contrast, IL‐1β produced moderate but consistent changes, including reduced staining intensity, increased histological degeneration relative to degenerative culture without cytokines, and trends toward increased matrix remodeling activity. These findings suggest that TNF primarily drives acute and rapid catabolic responses, whereas IL‐1β contributes to a moderate degenerative and matrix‐remodeling response. Such differences are consistent with established roles of these cytokines in cartilage biology, where TNF is associated with rapid apoptosis and matrix breakdown [74, 75, 76], and IL‐1β with regulation of proteolytic enzymes [77, 78, 79, 80].

Despite clear histological evidence of proteoglycan loss under cytokine stimulation, a hallmark feature of cartilage ECM breakdown [81, 82] and disc degeneration [83, 84, 85, 86], tissue‐level GAG/DNA ratios remained unchanged. This apparent discrepancy is explained by concurrent reductions in cell density, particularly in TNF‐treated samples, where loss of DNA offset reductions in GAG content. At the same time, elevated GAG levels in the culture medium suggested active matrix degradation. This dissociation between retained tissue GAG and increased extracellular release is indicative of an early‐stage degenerative process, in which matrix components are lost to the environment before complete depletion within the tissue [87, 88, 89, 90] and is consistent with previous studies showing that IL‐1β and TNF do not have a significant effect on the GAG content in the cartilage tissue [77]. Such patterns are consistent with early disc degeneration, where biochemical alterations precede structural disruption. The greater GAG release observed under TNF further supports its role as a potent catabolic driver, whereas IL‐1β appears to promote matrix turnover without equivalent matrix loss as seen in human studies [74, 78]. This difference may reflect interactions between cytokines and matrix components, as GAGs have been reported to modulate cytokine availability, although this mechanism requires further investigation.

Degeneration exhibited a distinct spatial pattern, with the CEP showing the earliest and most pronounced changes. Proteoglycan depletion, reduced staining intensity, and altered cellular organization were most evident in the CEP, while the BEP and soft tissues (NP/AF) showed comparatively limited changes over the same time frame. Quantitative histological analysis further demonstrated that reductions in staining intensity occurred without substantial loss of stained area, indicating that matrix composition was altered prior to structural disruption. Cytokine‐treated samples also showed increased chondrocyte clustering and reduced cell density, consistent with cellular stress and early degeneration. In contrast, collagen‐rich structures remained relatively preserved, suggesting that early degeneration primarily affects the non‐collagenous matrix. Together, these findings support a model in which degeneration in the CEP is characterized by early biochemical and cellular changes before structural breakdown becomes apparent.

A major strength of this study is the use of an intact multi‐tissue IVD–VB explant model that preserves the native bone–cartilage–disc interface. Tissue thicknesses of CEP and BEP across all conditions fell within established physiological ranges reported in the literature [4, 9, 10, 91, 92, 93, 94, 95] confirming structural consistency across samples. This uniformity strengthens the interpretation that observed degenerative changes are attributable to experimental conditions rather than baseline anatomical variability. Unlike previous models that rely on isolated cells or simplified tissue systems, this approach maintains continuity between the NP/AF, CEP, and BEP. This enables the investigation of cross‐tissue signaling and transport processes in a physiologically relevant context, which in turn complements the cross‐talk hypothesis previously mentioned [31, 32, 33, 34, 35].

Literature specifically addressing cytokine‐driven degeneration of the EP remains limited [4, 9, 10, 93, 94]. Most studies have focused on Modic changes or have used isolated CEP cells and simplified systems, which do not capture the complexity of the native tissue interfaces [96, 97, 98]. To date, there are only two other studies that have directly addressed cytokine‐induced degeneration in diced human CEP tissues. One related TNF to the activation of NF‐κB pathways and ADAMTS‐5 upregulation [99], and the other related IL‐1β to cell activity and cartilage‐related proteins [100]. While these findings align with the catabolic responses observed in the present study with bovine samples, they lack the structural and interfacial context preserved in the current model. By contrast, the current work investigates cytokine‐induced degeneration within an IVD–VB explant, providing a platform to study cytokine‐driven degeneration within an intact IVD‐EP interface, while enabling future investigation of potential inter‐tissue interactions in situ. Two other studies also used digested human CEP cells, but did not focus on cytokines; they investigated TGF‐β3 [101] and IGF‐1/CA12 [102] to enhance anabolic processes and prevent calcification.

Some limitations should be considered in this study. The culture system was static and did not incorporate mechanical loading, which plays a key role in disc physiology and EP function. Washing the samples could lead to changes compared to the in vivo conditions. The duration of culture was limited to 14 days, restricting analysis to early degenerative changes. Only single cytokine concentrations were evaluated, and additional cytokine mediators may contribute to disease progression, although these cytokines will likely induce further cytokine secretion from the tissues [39]. Furthermore, while bovine tissue provides a relevant structural model, species‐specific differences may influence cellular responses. Future studies incorporating mechanical stimulation, extended culture durations, and broader molecular analyses would further enhance the applicability of this model. It should also be noted that the *in silico* model, originally developed and validated in the context of osteoarthritic chondrocytes [36, 37, 38], simulates degenerative stress via low osmolarity, while the experimental design modulates pH as the primary degenerative cue, as the low osmolarity is modeled in both standard and degenerative media conditions. Although both parameters are characteristic of the degenerative IVD niche [50], and the directional predictions of the model were borne out experimentally, the two systems are not identical surrogates of one another. Future work should aim to integrate both osmolarity and pH perturbations within a single computational and experimental framework, and to establish whether the model's network topology adequately represents CEP chondrocyte biology, given known phenotypic differences between CEP and articular chondrocytes.

In conclusion, this study demonstrates that cytokines are key drivers of early EP degeneration, with TNF and IL‐1β exerting distinct but complementary effects on matrix degradation and remodeling. By integrating computational modeling with a structurally validated, multi‐tissue explant model, this work provides new insight into the mechanisms underlying EP pathology and establishes a physiologically relevant platform for investigating therapeutic strategies targeting cytokine‐driven disc degeneration.

## Author Contributions

**Ahmad Alminnawi:** conceptualization, methodology, validation, data curation, formal analysis, investigation, writing – original draft, visualization, writing – review and editing. **Pablo Tejero:** methodology, investigation, writing – review and editing. **Carla Geeroms:** methodology, investigation, writing – review and editing. **Liesbet Geris:** formal analysis, supervision, funding acquisition, writing – review and editing, project administration, resources. **Christine L. Le Maitre:** conceptualization, formal analysis, supervision, resources, project administration, funding acquisition, visualization, writing – review and editing.

## Funding

This work was supported by the European Commission (955735, 101088919, 101079372).

## Conflicts of Interest

The authors declare no conflicts of interest.

## Supporting information


**Table S.1:** Sample overview showing presence of nucleus pulposus (NP), annulus fibrosus (AF), or both (NP + AF) in the soft tissues (ST) of each sample stratified by bovine donor and culture medium. Day 0 (D0) signifies the start of the experiment, taking place 3 days post harvesting. Samples were maintained for 14 days in Standard or Degenerate medium, with cytokine‐treated groups denoted by “+ TNF” or “+ IL‐1β”. Sample numbers are indicated by “_#”.
**Figure S.1:** Normalized expressions of the (a) protein, (b) gene, and (c) global activity of the 74 parameters of the chondrocytes in the degenerate environment with and without cytokines compared to the reference healthy chondrocytes (healthy control) and reference degenerate chondrocytes (degenerate control).
**Figure S.2:** Representative μCT scans showing the visible growth plates of (a) bovine 1 and (c) bovine 3 vs. the closed growth plate of (b) bovine 2. The location of the growth plate is indicated by the translucent rectangle. Scale bar = 1 mm.

## Data Availability

The data that support the findings of this study are available from the corresponding author upon reasonable request.
