# Supplementary material for: Cytokine‐Mediated Responses Across Endplate Subregions in Intervertebral Disc Multi‐Tissue Explants
Source: JOR Spine. 2026 Nov 11;9(4):e70225. doi: 10.1002/jsp2.70225 (PMC13752716; doi:10.1002/jsp2.70225)
Supplement: Supplementary file 1 [file JSP2-9-e70225-s001.docx]

Table S.1: Sample overview showing presence of nucleus pulposus (NP), annulus fibrosus (AF), or both (NP+AF) in the soft tissues (ST) of each sample stratified by bovine donor and culture medium. Day 0 (D0) signifies the start of the experiment, taking place 3 days post harvesting. Samples were maintained for 14 days in Standard or Degenerate medium, with cytokine-treated groups denoted by “+ TNF” or “+ IL-1β”. Sample numbers are indicated by “_#”.

| **Bovine** | **Sample medium** | **ST type** |  | **Bovine** | **Sample medium** | **ST type** |
| --- | --- | --- | --- | --- | --- | --- |
| 1 | D0_1 | AF + NP |  | 2 | Degenerate_2 | NP |
| 1 | D0_2 | AF + NP |  | 2 | Degenerate_3 | NP |
| 1 | D0_3 | AF + NP |  | 2 | Degenerate + IL-1β_1 | NP |
| 1 | Standard_1 | NP |  | 2 | Degenerate + IL-1β_2 | NP |
| 1 | Standard_2 | AF |  | 2 | Degenerate + IL-1β_3 | NP |
| 1 | Standard_3 | AF + NP |  | 2 | Degenerate + IL-1β_4 | NP |
| 1 | Degenerate_1 | AF |  | 2 | Degenerate + TNF_1 | NP |
| 1 | Degenerate_2 | AF |  | 2 | Degenerate + TNF_2 | AF + NP |
| 1 | Degenerate_3 | AF + NP |  | 2 | Degenerate + TNF_3 | AF + NP |
| 1 | Degenerate + IL-1β_1 | AF + NP |  | 2 | Degenerate + TNF_4 | NP |
| 1 | Degenerate + IL-1β_2 | AF + NP |  | 3 | Standard_1 | NP |
| 1 | Degenerate + IL-1β_3 | AF + NP |  | 3 | Standard_2 | NP |
| 1 | Degenerate + TNF_1 | AF + NP |  | 3 | Standard_3 | NP |
| 1 | Degenerate + TNF_2 | NP |  | 3 | Degenerate_1 | NP |
| 1 | Degenerate + TNF_3 | NP |  | 3 | Degenerate_2 | NP |
| 2 | D0_1 | NP |  | 3 | Degenerate_3 | AF + NP |
| 2 | D0_2 | NP |  | 3 | Degenerate + IL-1β_1 | AF + NP |
| 2 | D0_3 | NP |  | 3 | Degenerate + IL-1β_2 | NP |
| 2 | Standard_1 | AF + NP |  | 3 | Degenerate + IL-1β_3 | NP |
| 2 | Standard_2 | NP |  | 3 | Degenerate + TNF_1 | NP |
| 2 | Standard_3 | NP |  | 3 | Degenerate + TNF_2 | AF + NP |
| 2 | Degenerate_1 | NP |  | 3 | Degenerate + TNF_3 | NP |

Figure S.1: Normalized expressions of the (a) protein, (b) gene, and (c) global activity of the 74 parameters of the chondrocytes in the degenerate environment with and without cytokines compared to the reference healthy chondrocytes (healthy control) and reference degenerate chondrocytes (degenerate control)

Figure S.2: Representative µCT scans showing the visible growth plates of (a) bovine 1 and (c) bovine 3 vs. the closed growth plate of (b) bovine 2. The location of the growth plate is indicated by the translucent rectangle. Scale bar = 1 mm.
